# The role of soil temperature in mediterranean vineyards in a climate change context

**DOI:** 10.3389/fpls.2023.1145137

**Published:** 2023-05-09

**Authors:** J. Miguel Costa, Ricardo Egipto, Francisca C. Aguiar, Paulo Marques, Amaia Nogales, Manuel Madeira

**Affiliations:** ^1^ Linking Landscape, Environment, Agriculture and Food, LEAF Research Center, Instituto Superior de Agronomia, Universidade de Lisboa, Lisboa, Portugal; ^2^ Laboratório Associado TERRA, Instituto Superior de Agronomia, Lisboa, Portugal; ^3^ INIAV, Instituto Nacional de Investigação Agrária e Veterinária, Polo de Inovação de Dois Portos, Dois Portos, Portugal; ^4^ CEF, Centro de Estudos Florestais, Instituto Superior de Agronomia, Universidade de Lisboa, Lisboa, Portugal; ^5^ Instituto Superior de Agronomia, Universidade de Lisboa, Lisboa, Portugal

**Keywords:** radiation, row-crops, sustainable soil management, thermal data, water, soil temperature sensing, cover crops

## Abstract

The wine sector faces important challenges related to sustainability issues and the impact of climate change. More frequent extreme climate conditions (high temperatures coupled with severe drought periods) have become a matter of concern for the wine sector of typically dry and warm regions, such as the Mediterranean European countries. Soil is a natural resource crucial to sustaining the equilibrium of ecosystems, economic growth and people’s prosperity worldwide. In viticulture, soils have a great influence on crop performance (growth, yield and berry composition) and wine quality, as the soil is a central component of the *terroir*. Soil temperature (ST) affects multiple physical, chemical and biological processes occurring in the soil as well as in plants growing on it. Moreover, the impact of ST is stronger in row crops such as grapevine, since it favors soil exposition to radiation and favors evapotranspiration. The role of ST on crop performance remains poorly described, especially under more extreme climatic conditions. Therefore, a better understanding of the impact of ST in vineyards (vine plants, weeds, microbiota) can help to better manage and predict vineyards’ performance, plant-soil relations and soil microbiome under more extreme climate conditions. In addition, soil and plant thermal data can be integrated into Decision Support Systems (DSS) to support vineyard management. In this paper, the role of ST in Mediterranean vineyards is reviewed namely in terms of its effect on vines’ ecophysiological and agronomical performance and its relation with soil properties and soil management strategies. The potential use of imaging approaches, e.g. thermography, is discussed as an alternative or complementary tool to assess ST and vertical canopy temperature profiles/gradients in vineyards. Soil management strategies to mitigate the negative impact of climate change, optimize ST variation and crop thermal microclimate (leaf and berry) are proposed and discussed, with emphasis on Mediterranean systems.

## 1 Introduction

### 1.1 European viticulture and climate change

Agriculture is a nature-based, climate-dependent sector and is strongly affected by climate change. A recent report from the European Environment Agency indicates that the overall impacts of climate change can decrease significantly the EU’s agricultural sector production (up to 16% loss in income by 2050), with large regional variations (EEA, 2019). Even in regions not experiencing a decrease in rainfall, air temperature rise will result in higher evapotranspiration (Seneviratne et al., 2010; Abad et al., 2021). For this reason, the agricultural sector must build up the capacity to adapt to increasing dry and warm conditions induced by climate change. Soil characteristics and soil management have a major role in this adaptation (EEA, 2019), but a better understanding is needed for Mediterranean viticultural systems.

The EU protects high-quality wines by linking them to legally defined geographic areas, specific sustainable production practices, traditional varieties and soil characteristics (Candiago et al., 2022; Onofre, 2022). The contribution of Mediterranean viticulture (e.g. Spain, Italy, France, Portugal and Greece) to the global wine industry is large, accounting for more than 50% of the world production and about 55% of world exports (OIV, 2022). However, Mediterranean viticulture is highly vulnerable to climate change (Costa et al., 2016; Fraga et al., 2016; Santos et al., 2020; Xyrafis et al., 2022), especially to the combination of longer warmer and drier periods. The same occurs for other Mediterranean perennial crops, such as olive groves and almond orchards (Andrade et al., 2014; Garcia-Tejero et al., 2018; Fraga et al., 2021).

Higher air temperature promotes earlier bud break, flowering, maturation and harvest, which can be negative for berry composition (e.g. higher sugar concentration and decreasing acidity) and can result in unbalanced wines (Bonada et al., 2015; Droulia and Charalampopoulos, 2022). Drier conditions exacerbate the effects of heat stress because dry soils cannot provide latent heat cooling by evapotranspiration, resulting in higher and more stressful temperatures at the plant level (Seneviratne et al., 2010; Stéfanon et al., 2014; Guion et al., 2022). This not only affects vine’s phenology but also yields and vines longevity and, ultimately the overall sustainability of the sector (economical, environmental and social) (Santos et al., 2020; Costa et al., 2022; Droulia and Charalampopoulos, 2022). In addition, these climatic scenarios may limit the expansion of the cultivated area in some regions of Mediterranean countries and may force the relocation of vineyards at higher altitudes (Jones, 2012).

### 1.2 Soil, climate change and surface energy balance

Soils are critical to sustain the equilibrium of ecosystems, economic growth and people’s prosperity worldwide (Brady and Weil, 2017). Soils provide multiple ecosystem services and socio-economic activities and in viticulture, they are an important component of the *terroir*, since they are one of the major factors influencing berry traits, wine characteristics and styles (White, 2015; Van Leeuwen et al., 2018; Sremac et al., 2021). Soils have a relevant function in the adaptation of the agricultural sector to adverse climatic conditions and more sustainable soil management is needed to ensure food security but also to improve adaptation to climate crises (EEA, 2019; Cataldo et al., 2021). Soil characteristics govern vegetation growth and influence heat, water and carbon fluxes between soil and the atmosphere (Evett, 2000; Lorenz et al., 2010; Liu et al., 2022).

**Figure 1:**
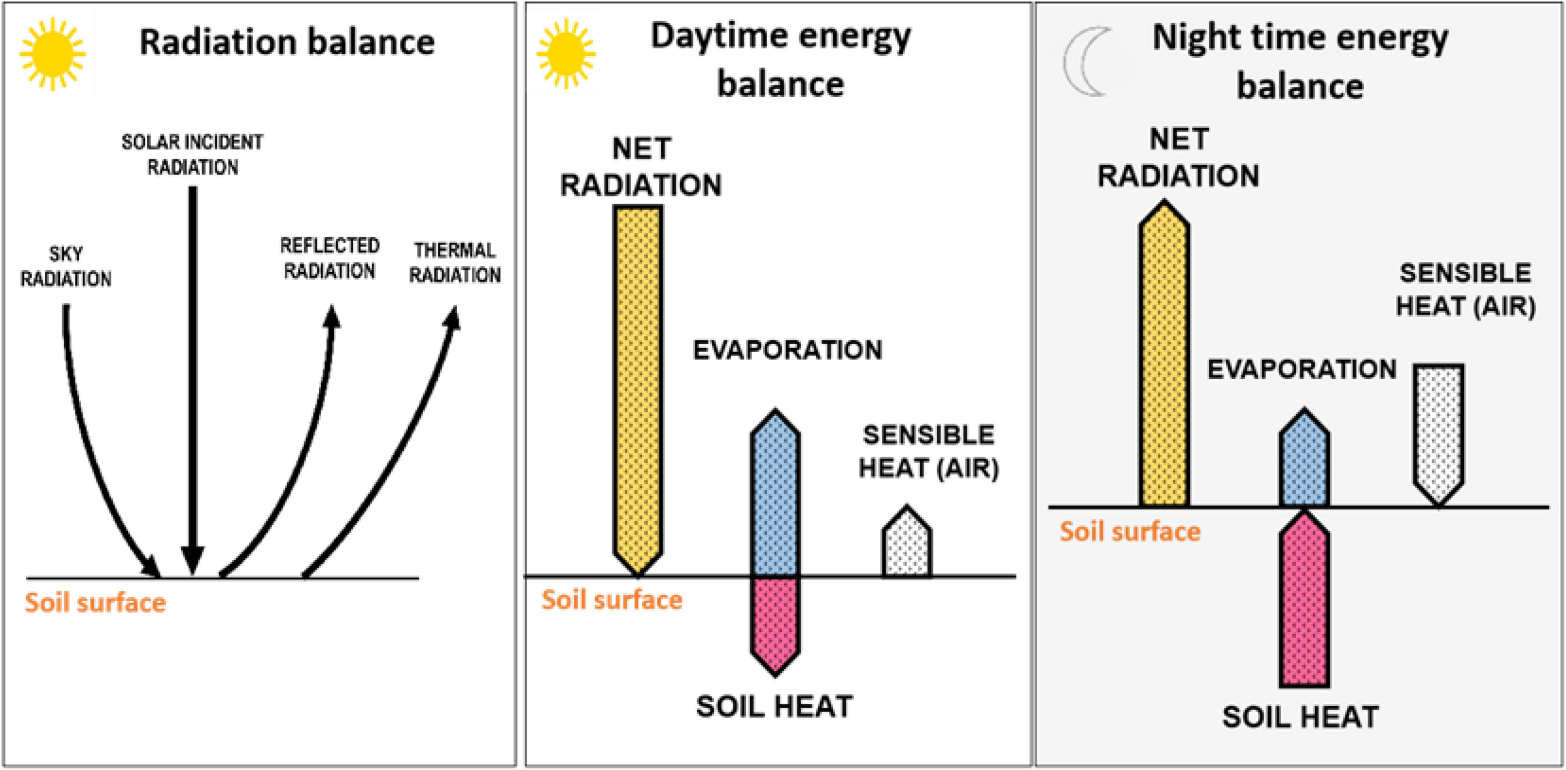
Schematic representation of the radiation balance, the daytime energy balance, and the night time energy balance. Net radiation at soil surface represents the sum “solar radiation plus sky radiation” minus the sum of “reflected radiation plus thermal radiation”. Most of the radiation that reaches earth’s surface in the daytime is used for evapotranspiration or reflected and emitted to the atmosphere. Evaporation translates the latent heat. The arrows indicate the direction of the exchange and arrow lengths tentatively indicate the magnitude of the different fluxes (Adapted from Evett, 2000; Hillel, 2004 and Brady and Weil, 2017).

Soil-atmosphere temperature relations are particularly important in the context of climate change (Hirschi et al., 2011). They involve partitioning of the surface energy into sensible (H) and latent heat (LE) fluxes **(** **Figure 1** **),** which depend on soil moisture content (Wang and Yang, 2018). Under dry conditions, the available net radiation (Rn) energy is converted into H fluxes, which increases air temperature. The relationship (coupling) between ST and soil moisture regimes explains the use of both variables in natural resource management, to better quantify and predict climate change impacts (Houle et al., 2012; Bradford et al., 2019). The energy balance equation for soil is commonly expressed as: Rn = LE + H + G, in which Rn is the net flux density of radiation (W/m^2^), and G is the soil heat flux,

Soil characteristics and soil management influence the energy balance at the soil’s surface and on the plant’s energy balance due to the reflection of shortwave irradiation that becomes a source of longwave radiation for plants (Nobel, 2005) (**Figure 1**). Furthermore, ST influences physical, chemical and biological processes taking place in the soil and regulates energy and matter exchange with the atmosphere (Baver, 1965; Hillel, 2004; Brady and Weil, 2017). Soil temperature influences evaporation, aeration and the type and rates of chemical reactions occurring in soils (Hillel, 2004).

Predictions for air temperature increase due to climate change are well described in the literature (IPCC, 2021). However, less information is available for ST. In a recent study, Schultz (2022) reports a progressive increase of ST for Northern European countries (e.g. Germany) in the last decades. Nonetheless, this trend observed for ST is expected to be more marked in Southern Europe. The Mediterranean region has a warm season transitional climate, in which evapotranspiration is limited by low soil moisture rather than by solar radiation (Feng et al., 2022). In this context, low soil moisture will amplify heat anomalies and extremes in the region (Seneviratne et al., 2010; Vogel et al., 2021; Feng et al., 2022) with a potential major impact on growth and yield of both crop and weed species.

In Mediterranean areas, ST and soil moisture regimes are classified as xeric, as precipitation concentrates in the winter and summers are dry, and the mean annual ST can range between 15 and 22°C (Soil Survey Staff, 2014). Soil temperature is one of the major drivers of grapevine physiology, growth and productivity. Soil temperature affects physical and biological processes at soil’s surface (e.g. weed and crop phenology, growth, respiration, etc.) (Bullied et al., 2003; Howell et al., 2020). At deeper soil layers ST influences root metabolism and growth, soil respiration, water and nutrient uptake, microbial diversity and activity, organic matter (OM) dynamics, soil bio-chemistry) (Akter et al., 2015; Onwuka and Mang, 2018; Mehdizadeh et al., 2020a; Mehdizadeh et al., 2020b; Shah et al., 2022).

Mean temperatures of air and soil, and in particular their extremes, influence weeds and crop physiology (seeds, fruits, leaves, and roots) (Bullied et al., 2003; Chaves et al., 2016; Field et al., 2020; Gambetta et al., 2021; Sremac et al., 2021). In vineyards, the proximity of leaves and clusters to soil surface enhances the warming effect of ST and soil sensible heat fluxes on berries, clusters and leaves. This is observed for the worldwide used Vertical Shoot Positioning (VSP) system in which the cluster zone and basal leaves often get warmer than the upper part of the canopy due to soil sensible fluxes, under warm and dry conditions (Costa et al., 2019).

A deeper understanding of vineyard soils, including their properties, functions, ecological roles, and management is required to increase the resilience of Mediterranean viticulture systems to more extreme climate conditions. There is a need to integrate the components of the *terroir* related to ST and the solutions for adaptation to climate change. This must be done at local level and should consider the trade-offs between adaptation strategies (Naulleau et al., 2021). In the following section, some of the major determinants of ST are presented.

## 2 Determinants of soil temperature in vineyards

Soil properties (e.g. color, texture, structure, moisture content) together with dominant atmospheric conditions (e.g. air temperature, solar radiation and wind) (Baver, 1965; Evett, 2000) influence soil heat and water fluxes and ultimately ST variables (thermal conductivity, thermal regime, maximum and minimum temperatures) (Brady and Weil, 2017). On the other hand, anthropological conditions, including agricultural soil management strategies (**Table 1**), can modify heat and water fluxes between soil, plant and atmosphere influencing ST variation (Neilsen et al., 1986; Rességuier et al., 2023).

**Table 1:** Non-exhaustive list of major determinants influencing soil temperature (ST), and general individual effect on the increase (↑), decrease (↓), reliable with other factors, such as climate (↓↑).

FACTORS	GENERAL EFFECT ON ST	SOURCE
Topographic		
Slope	flat↑ large sloping ↓	Baver (1965)
Exposition	North↓ South↑	Brady and Weil (2017)
Soil properties		
Color and albedo	dark ↑ light ↓	Meinhold et al. (2010)
Soil texture	silty ↑ sandy ↓	Sremac et al. (2021)
Organic matter content	OM and darker color↓ poor/lighter color↑	Brady and Weil (2017)
Soil structure	stable large round aggregates↓ unstable or platy, prismatic, blocky aggregates↑	Sremac et al. (2021)
Soil moisture	dry ↑ wet ↓	Brady and Weil (2017); Wang and Yang (2018); Krapez et al. (2012)
Soil and canopy management		
Tillage	↑	Radke (1982); Pradel and Pieri (2008)
Plant density	high ↓ low ↑	White (2015)
Canopy size/shedding	large ↓ small ↑	White (2015)
Irrigation	↓	Costa et al. (2019); Costa et al. (2020); Krapez et al. (2012)
Row orientation	↑ ↓	Hunter et al. (2021); White (2015); Pisciotta et al. (2021)
Soil cover^1)^	↓	Baver (1965); Lazcano et al. (2020); Akter et al. (2015); Brady and Weil (2017)
Soil and canopy cover^2)^	↓	Marigliano et al. (2022); Tadayon and Hossein (2022); Pradel and Pieri (2008)

^1)^mulching, natural vegetation, cover crops; ^2)^nets and other covering structures.

Extremes of the scale, when pertinent, are given as indicators of the general effect on ST.

**Figure 2:**
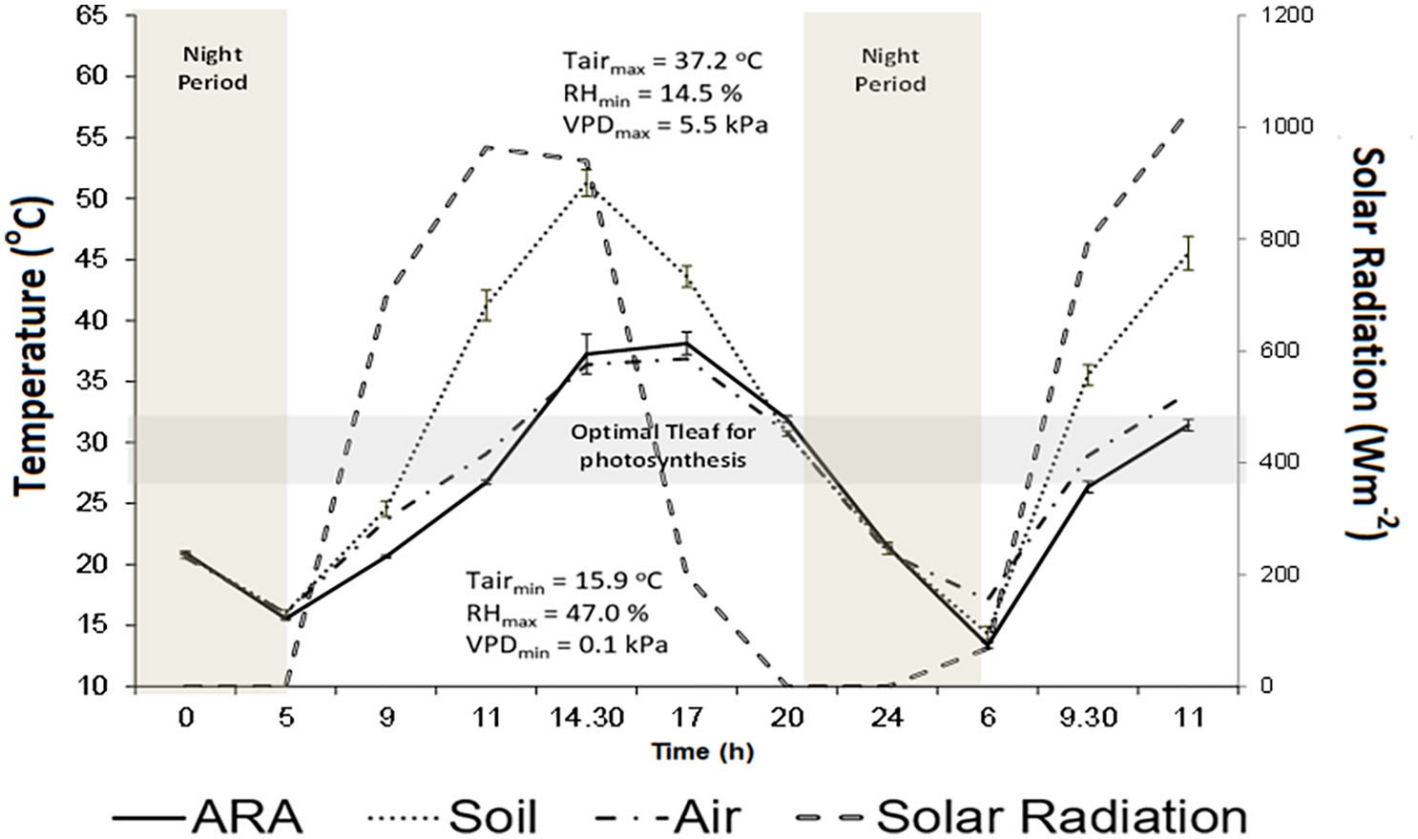
Diurnal variation of solar radiation (Wm^−2^) (– –), air temperature (T_air_), soil surface temperature (**^………^**, Soil) and vine’s canopy temperature (**^:_^** ARA) for the the *Vitis vinifera* cv Aragonez (syn. Tempranillo) (ARA), subjected to deficit irrigation, in a vineyard located in Alentejo (Southern Portugal). Data were collected along 8–9 July 2015 under the following climatic conditions (T_air min/max_ = 37.2 °C/15.9 °C; RH _min/max_ = 14.5%/47.0%; Wind speed _min/max_ = 0.6 m s^−1^/4.7 m s^−1^). Soil surface temperature was assessed by thermal imaging (Adapted from Costa et al., 2019).

The impact of climatic conditions on surface energy balance and consequently on ST is expressed by daily and seasonal variations in surface ST. In summer months (June–July in the northern hemisphere) maximum incident global radiation is closely related to maximum ST at midday (**Figure 2** **)** (Costa et al., 2019; Sremac et al., 2021). The stronger seasonal warming response of soils in summer as compared to autumn period mainly relates to decreased soil moisture content at the top layers of soil during summer (Schultz, 2022). Together with the effect of climate conditions, various soil properties and soil management (e.g. irrigation, mulching) can influence ST to a different extent (**Table 1**).

### 2.1 Topography and soil temperature

Soil temperature is related to the amount of incident radiation (**Figure 1**). Topographic components (e.g. slope and exposition to sunlight) influence ST and soil moisture regimes (Baver, 1965; Griffiths et al., 2009; Brady and Weil, 2017). Slopes with a southern aspect have higher levels of insolation, and consequently, higher heat accumulation and are usually considered ideal (Yau et al., 2014). Usually, the temperature of corrugated fields is higher than that of flattened ones due to different degrees of incident and reflected radiation (Brady and Weil, 2017). Radke (1982) in turn, reported that inclined ridge surfaces absorbed about 10% more solar radiation than flat surfaces contributing to higher ST.

### 2.2 Soil properties and soil temperature

#### 2.2.1 Soil albedo and color

The surface albedo represents the reflectivity of the Earth’s surface for incident solar radiation (Evett, 2000). The amount and type of reflected radiation depend on the characteristics of the surface and of the vegetation cover or mulch beneath crops, and soil properties (Meinhold et al., 2010). Soil vegetation controls the amount of sunlight that hits the ground surface, and bare soils cool down and warm up faster than soils covered with vegetation (Akter et al., 2015; Brady and Weil, 2017). Regarding soil color, dark-colored soils can warm more than light-colored soils, since they absorb more radiation (Baver, 1965). Nevertheless, large amounts of OM in dark soils can increase their water retention, which can offset the increased heat absorption due to the dark color (Brady and Weil, 2017). Soil’s albedo can be manipulated by different management strategies such as soil conservation tillage (Brady and Weil, 2017) or the use of certain products such as biochar (Verheijen et al., 2013), or the use of other organic (Burg et al., 2022) and inorganic mulches (Marshall et al., 1996; Aragüés et al., 2014).

#### 2.2.2 Soil texture and structure

**Figure 3:**
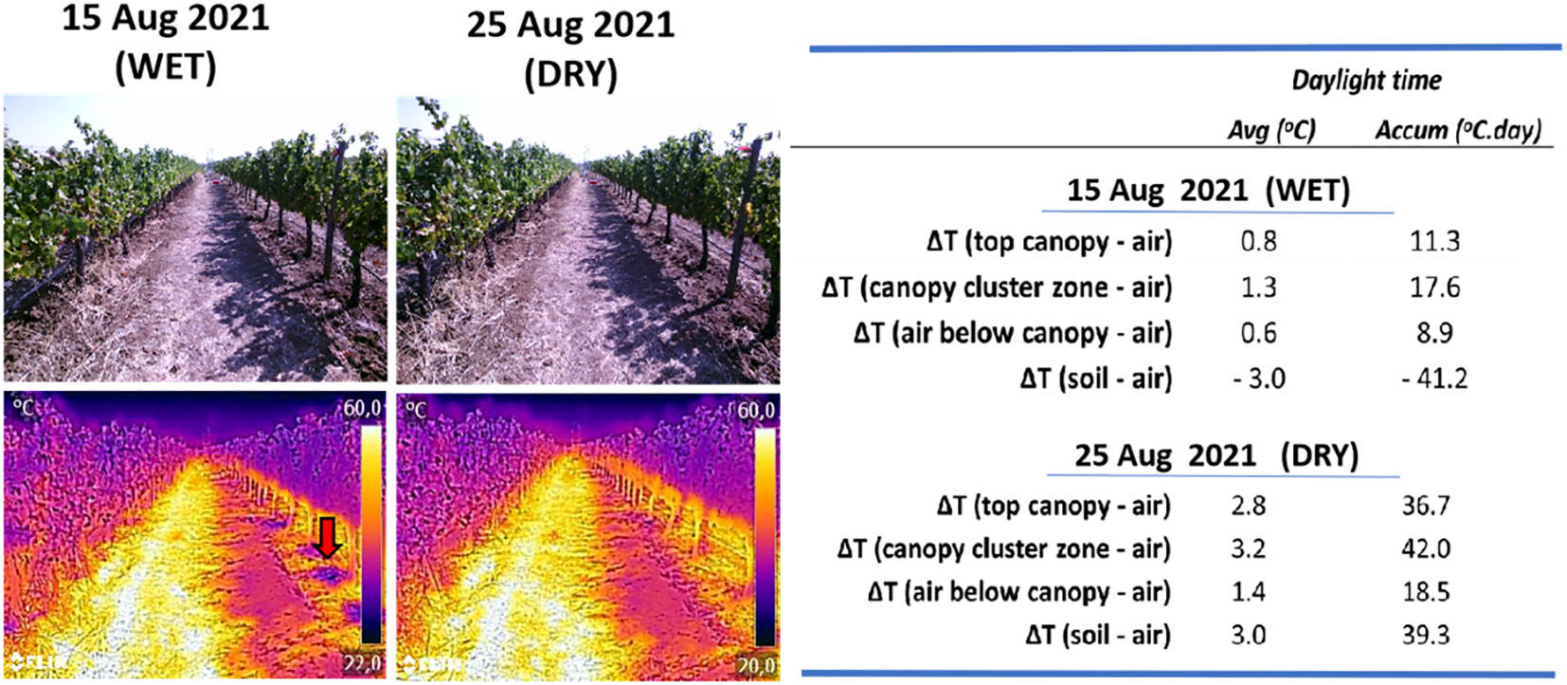
RGB and thermal images taken with a medium cost thermal camera (Flir C5, 160 x 120 pixels, 8-14μm, Emissivity = 0,96) from the inter row and rows with *Vitis vinifera* cv Tempranillo, taken at 16:00 hours, on 15 and 25 August 2021, showing the marked effect of shadow and sunlit soil sides as well as effect of irrigation on soil temperature (arrow) as part of a typical wet-drying cycle in irrigated vineyards. (Adapted from Egipto et al., 2022).

Soil texture refers to the proportion of sand, silt and clay sized particles that make up the mineral fraction of the soil, while soil structure refers to the organization of soil particles and the tendency of individual soil particles to combine into aggregates (Marshall et al., 1996; Hillel, 2004). The degree of aggregation influences water and air transport in the soil, solutes movement and soil’s biological activity (Brady and Weil, 2017). The texture influences soil thermal behaviour and soil surface temperature. Sandy soils tend to warm up faster than clay soils due to their lower heat capacity, lower thermal conductivity, and lower evaporative cooling (Hillel, 2004). On the other hand, the amplitude of the daily ST variation decreases in the order sand > loam > clay. Soil moisture at the surface and in the subsurface moderates the daily range of ST (Krapez et al., 2012) (**Figure 3** **)**. In orchards, sandy soils usually have a higher night time cooling rate than clay soils, due to a faster energy loss and lower minimum air temperature (Sremac et al., 2021).

Soil structure also influences ST. Soil structure controls pore spaces due to different arrangements of soil particles and soils with a more spherical structure warm up faster due to higher aeration and reduced waterlogging conditions (Brady and Weil, 2017). Soil structure is negatively affected by compaction which increases soil density and thermal conductivity which also enables faster changes in ST and affects root growth and morphology (Brady and Weil, 2017; Gürsoy, 2021).

#### 2.2.3 Soil water content

Soil water and heat fluxes are coupled (Wang and Yang, 2018) and their study is highly relevant for climate research (e.g. climate models) (Lanyon et al., 2004; Seneviratne et al., 2010) and agronomical and remote sensing research (Krapez et al., 2012; Kustas et al., 2022). The specific heat of water is higher than that of soil, and consequently, soils with high moisture have higher specific heat than dry soils, resulting in lower ST (Baver, 1965). Therefore, higher soil water content makes the variation (increase/decrease) in ST occur more slowly than in dry soils (Brady and Weil, 2017). Lower moisture content results in a higher conversion of solar radiation into sensible heat (measurable as temperature) **(** **Figure 1** **),** in opposite to high soil moisture conditions, in which the incident solar energy is used to evaporate water (Heilman et al., 1994). Soil water evaporation reduces ST, and the temperature difference between soil and the atmosphere is proportional to the evaporation rate (Zeng et al., 2021) and as a consequence soil surface temperature inversely correlates with soil water content (Krapez et al., 2012). As a result, the typical wet-dry irrigation cycles occurring in irrigated crops often result in spikes in ST (See **Figure 3** **).**

Severe precipitation events and flooding can greatly affect soil characteristics, leading in general to soil erosion, compactation and nutrient runoff, with detrimental effects on crops (root growth, yield) and soil fauna, and influencing soil temperature (Mancuso and Shabala, 2010; Ruperti et al., 2019).

#### 2.2.4 Soil organic carbon content

Soil organic carbon (OC) content depends on the balance between carbon inputs and outputs. Carbon inputs relate to plant productivity, while carbon outputs relate to microbial decomposition of OM. Soil OM decomposition is controlled by ST during wet periods and by the combined effect of soil water and ST during dry periods (Yuste et al., 2007). On the other hand, high ST promotes OM mineralization rates and drives several physical, chemical and biological changes, which accelerates microbial decomposition of soil OM and, decreases soil fertility (Guoju et al., 2020). Soil microbial respiration uses soil OC and releases CO_2_, and higher ST promotes soil respiration and higher CO_2_ release to the atmosphere (Karhu et al., 2014).

Soil management practices (tillage, the use of cover crops, mulching) combined with changes in soil water content due to precipitation or irrigation, influence C dynamics in soils and soil biodiversity (Haddaway et al., 2017; Crystal-Ornelas et al., 2021). Soil tillage promotes CO_2_ release and disrupts protected OM in soil aggregates, increasing its availability for microorganisms (Haddaway et al., 2017). Precipitation and irrigation modify CO_2_ fluxes in soil, and the wet-dry cycles due to precipitation or irrigation events result in marked fluctuations in soil CO_2_ efflux and in dynamic responses in soil C pools (Yu et al., 2022).

## 3 The impact of soil temperature in vineyards

### 3.1 Grapevine responses

*Vitis vinifera* is a crop species well adapted to dry and warm conditions. However, more variable and extreme climatic conditions (heat and drought) pose risks to the wine sector. Temperature is a primary environmental factor influencing grapevine development, growth and physiological processes occurring in roots, shoots/leaves and berries, including growth and phenology, respiration and photosynthesis, flowering and fruit set, yield and berry composition (Chaves et al., 2016; Pagay and Collins, 2017; Field et al., 2020; Bernardo et al., 2021; Gambetta et al., 2021; Keller et al., 2022). Warmer conditions lead to earlier bud break and earlier harvests, resulting in wines with lower organic acids, higher pH levels, higher ethanol levels and altered sensory characteristics (Van Leeuwen and Destrac-Irvine, 2017; Venios et al., 2020). Heat stress due to air temperatures above 35°C decreases the synthesis of secondary metabolites, reduces photosynthesis rates and vegetative growth (Moutinho-Pereira et al., 2004; Chaves et al., 2010) and may affect plant water transport (Galat Giorgi et al., 2020). Excessively high temperatures (air and soil) in wine-growing regions can reduce berry color due to inhibition of anthocyanin biosynthesis or their degradation and promote the synthesis of reactive oxygen species (ROS) (Carvalho et al., 2014; Venios et al., 2020).Higher average air temperatures in the growing season can negatively impact yield and quality (Bonada et al., 2015) and brief episodes of extreme temperatures are detrimental when occurring at specific phenological stages, e.g. flowering and fruit set (Pagay and Collins, 2017; Gambetta et al., 2021; Keller et al., 2022).

High air temperatures and drought stress can influence leaf morphology and structure resulting in larger but thinner leaves, with smaller cells, and higher stomatal density (Pierce et al., 2022). High diurnal air temperatures and low night air temperatures ensure a low pH in berries which is highly relevant for wine production in warm areas (e.g. Mediterranean), which are increasingly experiencing an increase in night time temperatures (Venios et al., 2020).

The response of grapevine to heat and drought stress depends on several factors that include the atmospheric climatic conditions, the genotype (variety/rootstock), soil characteristics and soil and crop management (Lopes et al., 2011; Bota et al., 2016; Simonneau et al., 2017). Leaf gas exchange traits (e.g. photosynthesis, transpiration or stomatal conductance) respond fast to abiotic stresses, namely to drought and high temperatures (Chaves et al., 2010; Simonneau et al., 2017). Optimal leaf temperatures for photosynthesis range between 25 and 30° C (Greer and Weedon, 2012), but stomatal response to the environment depends on the genotype and their strategy (isohydric or anisohydric) to cope with water stress while optimizing thermal regulation (Chaves et al., 2010; Chaves et al., 2016; Simonneau et al., 2017).

Current studies on crop response to high-temperature stress are mainly focused on the effects of air temperatures on the aerial part of plants/crops (shoots, leaves) and its immediate environment, while the potential adverse effects of high ST are less examined (Dong et al., 2016; Costa et al., 2019). This applies to the effects of day time and night time temperatures under scenarios of warmer nocturnal air temperatures that tend to increase root-zone ST (Dong et al., 2016).

Metabolic processes such as respiration, photosynthesis and transpiration are sensitive to short-term temperature fluctuations and air and ST influence carbohydrate relations in grapevine (Chaves et al., 2016; Venios et al., 2020; Gambetta et al., 2021). Carbohydrate reserves are most abundant in grapevine roots, and ST regulates their mobilization to shoot and trunk (Rogiers et al., 2011). Soil warming up to 24°C promote shoot growth by increasing the use of starch reserves, while soil cooling (13°C) result in starch accumulation in both roots and stem and shift the overall biomass partitioning to the root system (Field et al., 2020). High night air temperature reduces carbohydrates exportation from leaves, but promotes respiration resulting in lower leaf carbohydrate contents (Tombesi et al., 2019). Moreover, higher air temperatures coupled with higher surface ST during early evening may promote excessive carbon loss due to maintenance of higher respiration (Escalona et al., 2012). Therefore, carbon losses and modified carbohydrate dynamics due to increased respiration must be better quantified for vines growing under dry and warm conditions (Medrano et al., 2016).

In grapevine, pot-based experiments showed that the highest biomass production and shoot growth rates were achieved under warmer treatment regimens (24°C compared to 13°C) (Field et al., 2020). However, above certain critical temperatures, growth is often hindered due to lower net assimilation rates, and in more extreme cases leaf overheating and death (Chaves et al., 2016). Supra-optimal ST may also affect the root system. Root survival can be negatively affected by ST above 35°C, which can lead to root death (Huang et al., 2005). Fluctuations in air temperature and ST modulate grapevine’s morphology. ST can affect root characteristics (size, architecture, and function) (Luo et al., 2020; Gavelienė et al., 2022). Exposure of plant roots to temperatures above their optimum often decreases primary root length and lateral root density, reducing the volume of soil explored by roots and consequently, reducing water and nutrient uptake (Koevoets et al., 2016). Meanwhile, the root architecture may dynamically adapt to spatial and temporal temperature changes by acclimation of root structure and geometry (Nagel et al., 2009; Fonseca de Lima et al., 2021; Fichtl et al., 2023). Nevertheless, it is difficult to quantify the effect of root temperature in real conditions, as the distribution of roots changes according to soil characteristics and conditions of the soil surface (Pradel and Pieri, 2008).

Temperature influences grapevine hormonal relations at root and shoot level (Walker and Winter, 2006; Chaves et al., 2016; Field et al., 2020; Bernardo et al., 2021). Soil temperature was found to regulate hormone content such as cytokinins (CKs) in grapevine xylem sap (Field et al., 2020) and also of abscisic acid (ABA) (Bernardo et al., 2021).The effects of ST on vine performance still need to be better quantified and the interaction between air and ST and soil moisture on vines must be evaluated for their physiology and agronomical performance under extreme dry and high-temperature conditions

### 3.2 Vineyard weeds and spontaneous vegetation

Soils of vineyards in the Mediterranean region are often subjected to intensive labor to reduce or eliminate competition by light, water, and nutrients, between vines and the weedy flora. Therefore, vineyard landscapes depend on a great investment in tillage, mowing, or herbicide application (Winter et al., 2018). Intensive soil management practices result in increasing ST with feedback loops on soil seed bank, altered seed dormancy, seed longevity and germination patterns, along with general plant composition changes towards resilient species to heat and water stress (Kathiresan and Gualbert, 2016). ST and soil moisture are key determinants for seed dormancy breaking and a trigger for germination, along with exposure to flashes of light on non-deep buried seeds caused by soil disturbance (Sauer and Struik, 1964). Higher ST due to bare soils and warmer climate conditions can promote synchronized mass seed germination of certain species, resulting in homogeneous and well-adapted weedy plant communities, which can be more damaging if agrochemicals (fertilizers and pesticides) are used, stimulating growth and weed resistance to herbicides. However, the mechanisms and traits of weed species can differ, and for some summer annual species, dormancy breaking occurs under low ST conditions, whereas optimal germination is trigged by higher ST (Forcella, 1998). Soil growing degree days (GDD) has been used with success for weeds, estimated from ST to predict emergence rates of weed seedlings. Following germination, higher air temperature and ST usually favoured rapid development of weed seedlings and plant growth, and GDD based on air temperatures can be used to estimate post-emergence seedling height growth (Forcella, 1998).

The proportion of bare soil was tested as a predictor of the taxonomic diversity of plant communities and vine yield, and results pointed to slightly lower berry productivity for higher plant diversity, corresponding to lower bare soil area, and lower ST (Guerra et al., 2022). Nevertheless, soil management decisions made by winegrowers involve cost-effectiveness of weed control that needs to be addressed locally and over the long term. These factors include, for instance, the weed resistance to herbicides, the role of weeds as a refuge for pests and diseases, or as resources for pollinators and pest predators, amongst other goods and services that spontaneous flora can provide to the well-being and society (Paiola et al., 2020). Higher ST promotes the dominance of exotic weedy species from tropical climates, such as *Conyza* spp. or native Mediterranean species that have clear positive photoblastic germination mechanisms, such as *Dittrichia viscosa* (Parolin et al., 2014). Evolutionary mechanisms of exotic annuals or seed-dispersed perennials out of the native range are likely to take place as an environmental adaptation, which increases the risk of unbalanced agroecosystems (Clements and Ditommaso, 2011; Garcia et al., 2018).

Effects of extreme ST on plants ecophysiology were studied for a few species and mostly on crops or grasslands but in general, extreme high ST affected photosynthesis by reducing carboxylation efficiency, with differences between C3 and C4 plants (Nóia Júnior et al., 2018). A reduction in leaf stomatal conductance, relative water content and increased concentration in intercellular CO_2_ occurred and C4 plants are likely to be more affected than C3, given the differences in photosynthetic pathways. In turn, extreme low ST on C4 plants resulted in higher leaf stomatal resistance and reduced photosynthetic rates.

Weeds and spontaneous vegetation present diverse seasonal dynamics that, together with the vine’s phenology, produce a dynamic ecosystem across time and space, on the rhizosphere and above ground. Relations with ST and vineyard management must be addressed by looking at the seasonality of the complex of crop-spontaneous vegetation and weedy flora, and the constraints and objectives of wine producers (Garcia et al., 2018).

### 3.3 Soil organisms

The biological component of the soil is a vital part of agricultural ecosystems, including vineyards, and is composed of a diverse set of macro- and micro-organisms like insects, myriapods, worms, nematodes, bacteria, archaea, fungi, actinomycetes, protozoa, algae (Pritchard, 2011; Sassenrath et al., 2018). They compose the soil food web and can be divided into four groups according to their body size and functional roles: micro-organisms and macro-, meso- and micro-fauna (Giffard et al., 2022). Those organisms have important ecological functions and lead crucial processes in the soil that determine soil and plant health, such as OM decomposition, nutrient cycling, soil structure improvement, including soil aeration and increased water and nutrient retention, as well as pest, disease and weed control (Giffard et al., 2022). Climate change and increased ST are likely to affect their diversity and community dynamics, which may have a strong influence on the overall food soil web and on the ecosystem services they provide, which may ultimately affect grapevine performance.

#### 3.3.1 Soil microbiota

In the particular case of soil microbiota, a rich and diverse community of soil microorganisms can ensure productive soils, because they largely influence nutrient cycling and soil fertility, promote pathogen suppression, enhance CO_2_ sequestration and increase soil OM mineralization rates. Due to those crucial roles for agro-ecosystem functioning, microbial biodiversity is considered as an important determinant of the *terroir* (Gobbi et al., 2022).

In vineyards, soils are the main reservoir of microorganisms for the grapevine phyllosphere and endosphere, since every growing season, aboveground plant organs, including leaves and berries, obtain their microbes mainly from the soil (Compant et al., 2011; Zarraonaindia et al., 2015). Nowadays, it is well accepted that balanced grapevine–associated microbial communities are essential not only for plant growth and biotic and abiotic stress tolerance (Pinto et al., 2014), but affect the organoleptic properties of the must (Zarraonaindia et al., 2015). It can also influence the fermentation dynamics at the winery and ultimately wine quality (Belda et al., 2017). Hence, any changes in soil microbial community composition and structure may influence plant performance and berry composition, given their direct and indirect influence on grapevine growth, health, stress tolerance and berry development (Di Giacinto et al., 2020).

Temperature is one of the most important determinants of microbial growth and metabolic rates. However, the assessment of the overall soil microbial community response as a function of temperature is still challenging (Jansson and Hofmockel, 2020) and an acclimatization of microbial communities to soil warming cannot be excluded (Pritchard, 2011; Snyder and Callaham, 2019). The increase of ST may have two contrasting consequences on soil organisms and microbiota. Since there is a linear relationship between temperature and respiration, it could be expected that in response to temperature rises, soil microbial respiration will also increase, releasing CO_2_ to the atmosphere (Bond-Lamberty et al., 2018), which contributes to the greenhouse effect, and further temperature increase. On the other hand, at higher temperatures, the metabolism of OM decomposers (mainly fungi) is expected to be more active (Schindlbacher et al., 2011) and, therefore, microbial OM mineralization rates could be faster. Under such circumstances, nutrient release is accelerated (e.g. nitrogen), which can be translated into faster plant growth, contributing to carbon sequestration. This issue is even more complex, with several other interacting factors in the local soil environment (e.g. high temperatures also affect the nitrification process or soil oxygen concentration) that ultimately affect microbial growth and metabolism (Bai et al., 2013; Hu et al., 2016; Jansson and Hofmockel, 2020).

Some studies demonstrate that as temperature increases, population shifts, and variations in microbial community structure and changes in functional genes occur (Zhang et al., 2005; Schindlbacher et al., 2011; Melillo et al., 2017; Romero-Olivares et al., 2017). In the case of vineyards, in a recent study across the world, Gobbi et al. (2022) found a positive correlation between temperature and fungal alpha diversity, but not between prokaryotic alpha diversity and temperature, indicating that fungal communities might be more sensitive to temperature than soil bacteria and archaea. Větrovský et al. (2019) also found that among the different environmental factors (climatic, soil and vegetation parameters), temperature and precipitation were the key factors regulating fungal diversity and community composition in soils. Nevertheless, to our knowledge, no studies have been conducted yet in vineyards to assess the direct effect of an increase in ST in key microbial soil processes as well as in the community composition/structure of soil micro-organisms. In particular, due to the major roles of fungal communities in vineyard soils as OM decomposers, as plant mutualists (and therefore promoting plant tolerance to stress factors) and also as pathogens, more knowledge is needed on the specific ways in which ST affects these communities. This will allow to develop better strategies to manage vineyard soils to buffer the negative effects of climate change on particular soil microbial functional groups.

#### 3.3.2 Soil fauna

Soil macro, meso and micro-fauna have important roles in the soil. They are involved in OM decomposition, attract microbial communities that mineralize nutrients, and contribute to improve soil structure by creating aggregates and soil pores and mixing the soil (Culliney, 2013). For instance, macroinvertebrates like earthworms, ants and thermites can move large portions of soil, creating new microhabitats for other soil organisms, and can assimilate plant materials, integrating them into the soil as OM (Snyder and Callaham, 2019). On the other hand, isopods and myriapods promote OM decomposition by feeding on carbon-based compounds and by excreting enzymes and feces into the soil, thereby enhancing the proliferation of microbial decomposer communities, that release nutrients into the soil (Hendrix, 2000; Zagatto et al., 2021).

The study of how soil warming affects soil fauna is challenging, since depending on the methodological approach (air, soil or air and soil warming), the outcome can be substantially different (Snyder and Callaham, 2019). Therefore, artificial/experimental temperature manipulation may not lead to a real response of soil fauna under natural conditions (Snyder and Callaham, 2019), which makes drawing conclusions somewhat challenging. Moreover, ST and moisture are directly linked, and therefore, differentiating the independent effects of each factor on soil fauna is often difficult. In addition, distinct taxonomical or functional groups may react differently to ST increases and to the indirect effects that this entails in the soil ecosystem.

In a model described by Snyder and Callaham (2019), the increase in ST leads first to changes in animal behavior, such as up- or downward movements in the soil profile. It can also lead to physiological changes that have consequences on their fitness and reproduction, with a subsequent effect on soil animal taxa abundance, community structure and diversity. This can ultimately lead to changes in their functions, including nutrient cycling, OM decomposition and soil respiration. In their review, Snyder and Callaham (2019) also present a conceptual model generalizing how soil warming may affect the soil environment and summarize the direct and indirect interactions that may occur between vegetation, microorganisms, soil macro-, meso- and microfauna and OM under a global warming context.

Although some studies already describe the diversity and ecosystem functions of soil fauna in vineyards (Ghiglieno et al., 2020; Gonçalves et al., 2021; Andrés et al., 2022; Giffard et al., 2022), much less information is available on the effects of soil warming on soil fauna (Snyder and Callaham, 2019). It is known that agricultural soils, and thus vineyards, tend to have low soil invertebrate diversity, which are often characterized by species that are well-adapted to environmental disturbances (Callaham et al., 2006). However, the management regime can strongly influence their populations, with a generally beneficial effect observed in arthropod abundance and diversity in organic vineyards (Caprio et al., 2015; Gagnarli et al., 2015; Ghiglieno et al., 2019; Ghiglieno et al., 2020; Bosco et al., 2022). According to Blankinship et al. (2011), soil fauna may be more sensitive to soil moisture content, and therefore to changes in precipitation, than to mild increases in temperature.

In a study conducted by Ghiglieno et al. (2020) in Italian vineyards, Collembola and Acari were the most frequent taxa observed in vineyard soils. While taxa of the first group showed a variety of responses related to ST, Acari, as well as Thysanoptera, Diplopoda and Hymenoptera were more related to lower ST. Contrastingly, Diptera, Isopoda, Hemiptera, as well as Coleoptera and Diptera larvae taxa abundance, was related to higher ST. This may lead to the hypothesis that the latter taxa may be more negatively affected by soil warming in the context of climate change than the former group, which could be benefited, since they appear to be thermophilic (Eisenbeis and Wichard, 1987; Reddy and Venkataiah, 1990; Zhu et al., 2018). This would be the case of ants, as some studies showed that they increase their activity at higher temperatures (Dunn et al., 2009; Snyder and Callaham, 2019).

More knowledge is needed on how soil fauna, in particular invertebrates, will react to soil warming and the associated changes in the above-ground vegetation and soil moisture. Understanding how those animal communities will respond to increased ST may help to decide on the most appropriate vineyard soil management strategies that can buffer ST changes and foster the proliferation of taxa that benefit both soil and crops.

## 4 Soil temperature measurements

Ecological patterns and processes are often more related to below-canopy soil temperature rather than to well-ventilated air temperatures (Lembrechts et al., 2022. Moreover, near-surface, rather than air temperature can work as better predictors of ecosystem functions and processes such as OM decomposition, soil respiration and other components of the global carbon balance (Lembrechts et al., 2022). Therefore, ST measurements are highly relevant and needed to achieve good reference data for specific ecological conditions as well as to use ST as a major variable to support modelling of ecosystem processes. However, due to the complexity and large labour costs of ST measurements, *in situ* observations of ST are less commonly described in literature than those of precipitation and air temperature (Li et al., 2020).

Soil surface temperature measurements are usually carried out with thermocouples and radiometers, but these devices have limitations concerning logistics, access, and technician costs (Frodella et al., 2020). In turn, measuring ST at different depths can be done with different types of thermometers and sensors installed at various depths (Abdel-Ghany et al., 2022), which do requires knowledge and significant manpower.

Consequently, there is a general consensus about the need to achieve soil spatial information (e.g. temperature, moisture) faster and with fewer human resources. The use of remote sensing technologies can offer an alternative or a complementary solution for localized and punctual measurements as it allows to retrieve a larger set of spatial data to study vegetation or soil properties at different resolution scales (temporal and spatial) (Jones and Vaughan, 2010; Wulf et al., 2014). In the last decades, it has increased the interest in developing methodologies for remotely measuring soil surface and vegetation temperature and to assess soil moisture conditions by using spaceborne, airborne or ground-based sensors (Jones and Vaughan, 2010; Krapez et al., 2012; Frodella et al., 2020; Xu et al., 2020; Diago et al., 2022).

Thermal imaging emerged as a highly flexible and non-contact measurement technique that enables small to large scale, surface temperature sensing and it can be used as an alternative or as a complementary tool for conventional soil surface temperature and moisture monitoring technologies, in a wide variety of geo-environmental and agricultural applications (Jones and Vaughan, 2010; Frodella et al., 2020; Zeng et al., 2021; Diago et al., 2022). Ground-based thermal imaging sensors, such as thermal cameras, experienced a fast technological development (e.g. focal plane array uncooled microbolometer sensors) that increased detectors’ accuracy, spatial resolution, and decreased costs (Jones and Vaughan, 2010).

Thermal imaging was successfully used in viticulture to monitor canopy and ST variation at different time scales and different irrigation conditions (Jones et al., 2002; Gutiérrez et al., 2018; Costa et al., 2019; Gago et al., 2020; Diago et al., 2022; Kustas et al., 2022) (**Figure 3** **)**. Thermography has been used to monitor the effects of soil, irrigation and different soil covering materials on ST in vineyards (Frodella et al., 2020). Other studies using thermography helped to characterize the mechanisms behind soil desiccation cracking (Zeng et al., 2021), or to study heat transfer processes in vineyards (Kustas et al., 2022). Thermography has been also used as an alternative method to monitor and detect soil microbial activity (Schwarz et al., 2021).

Satellite remote sensing has been developed for thermal applications, but data calibration and validation remain complex and costly (Frodella et al., 2020). Indeed, there are still limitations concerning image resolution because satellite measurements still have limited spatial and temporal resolution (Basurto-Lozada et al., 2020), when considering their practical aplication to field crops. A typical vineyard canopy architecture and row disposition, characterizes by a large amount of bare soil/cover crop separating rows of trellised vines, which demands higher imaging spatial resolution (of one meter or less) for high robustness (Basurto-Lozada et al., 2020). Nevertheless, a combination of ground-based *in-situ* measurements with aerial and satellite imaging may be a solution to monitor ST more effectively (Xu et al., 2020). The same applies to methodological approaches based on the fusion of information retrieved from thermal and multispectral sensors to generate estimates of ST by using computational intelligence models (Basurto-Lozada et al., 2020).

Other techniques such as soil resistivity measurements can be used as a proxy for ST: soils with high resistivity have generally coarse-textured and are warm in contrast to low resistivity soils that are richer in clay and are cooler (Van Leeuwen et al., 2020). Soil electrical conductivity (or its reciprocal soil electric resistivity) reflects a combination of soil mineralogy, salts, moisture and texture, which makes it a robust parameter to characterize soil properties. The advantage of this proximal sensing methodology gives high-resolution maps of the soil resistivity, which can be further related to ST. Furthermore, regression equations have been developed to predict and map moisture content, topsoil thickness, and clay content (Samouëlian et al., 2005).

The development of digital soil science, that is the study of soil using the tools of the digital convergence (Wadoux and McBratney, 2021), also opens new possibilities for imaging studies applied to ST and their effects on plants and soil. In addition, the existing cooperative works and data sources on ST (Lembrechts et al., 2022) can open new opportunities to use ST data in agriculture.

## 5 Strategies to manage soil temperature in vineyards

**Figure 4:**
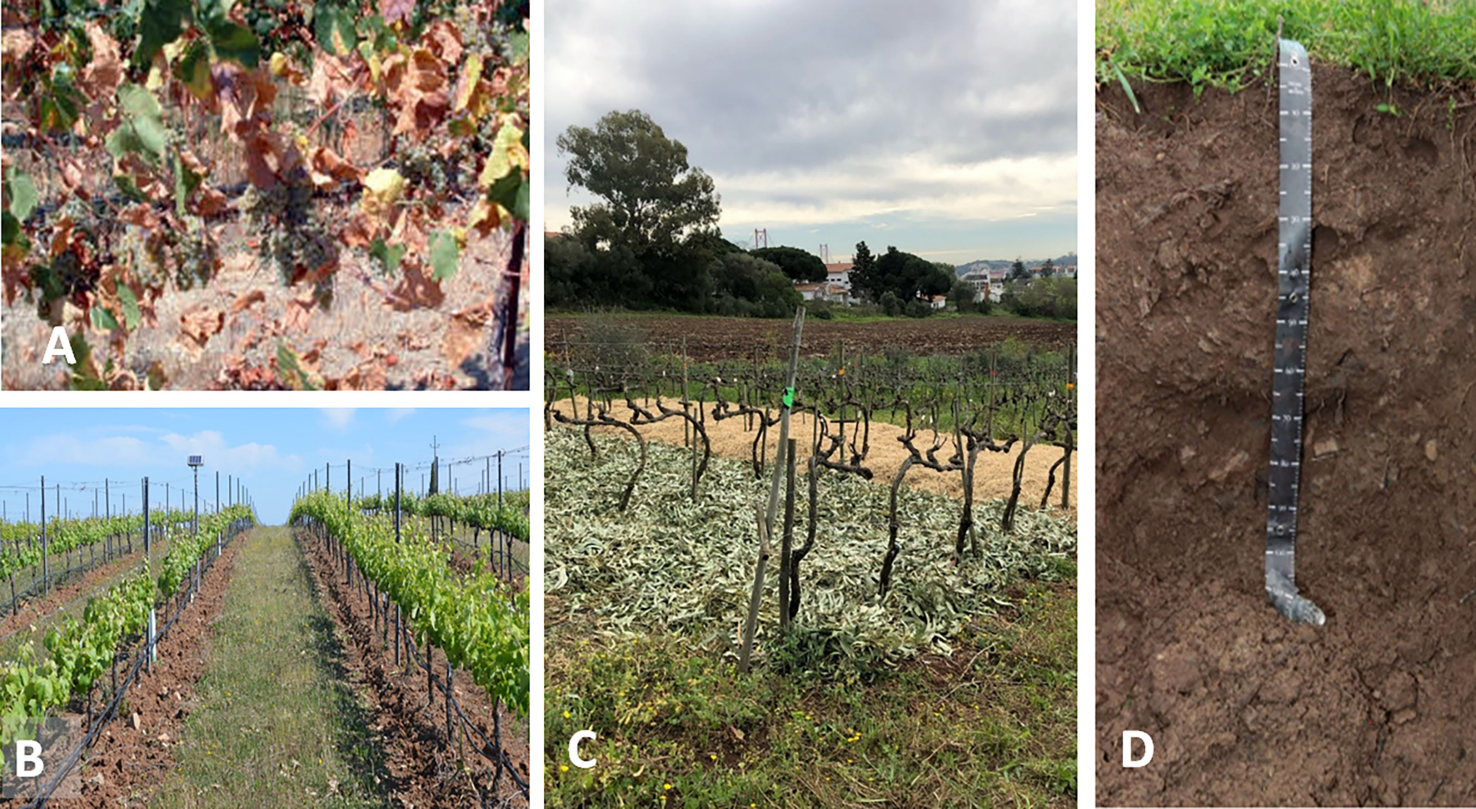
**(A)** The impact of dry and extreme heat in the basal leaves of a Mediterranean vineyard (South Portugal) and sustainable management practices in Mediterranean vineyards, **(B)** Soil grass cover in the inter row combined with row tillage in a vineyard in Alentejo’s wine region (South Portugal), **(C)** Mulching with different organic materials (rice straw and *Eucalyptus* foliage) and **(D)** Soil profile characterization as a tool to support best practices in soil management (ISA campus U. Lisboa).

Sustainable water and soil management are the core of several sustainability programs in the wine sector (Costa et al., 2022) and other perennial woody crops, such as olive groves and almond orchards (Andrade et al., 2014; Garcia-Tejero et al., 2018; Fraga et al., 2021). More sustainable practices related to soil management can help to alleviate the harmful effects of more extreme drought and heat events due to climate change. This is an increasingly important issue for Mediterranean viticulture and must combine effective soil and canopy management strategies **(** **Figure 4** **),** together with more efficient use of irrigation water and better-adapted varieties/rootstock combinations (Andrade et al., 2014; Costa et al., 2016; Cataldo et al., 2021; Naulleau et al., 2021).

**Figure 5:**
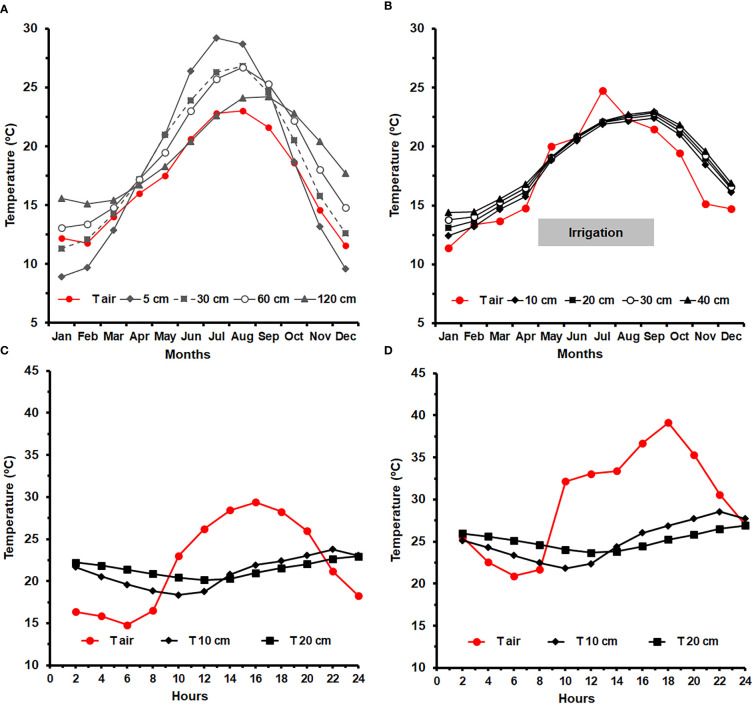
**(A)** Annual variation of soil monthly mean temperature (ST) at 5, 10, 30, 60 and 120 cm depth, for a 30-yr period (1931-1960), measured at the campus of the Instituto Superior de Agronomia (ISA), Lisbon (38°42’27.5’’N; 9°10’56.3’’W), under rainfed conditions (Botelho da Costa, 1995). **(B)** Monthly variation ST during the year of 2022, at 10, 20, 30 and 40 cm, in an irrigated vineyard, at ISA (data provided by Hidrosoph, Oeiras, Portugal). The red line indicates variation in the monthly mean air temperature, and the peak in July relates to a heat wave event; Daily ST variation during 1^st^ July 2022 **(C)** and 9^th^ July 2022 **(D)**, respectively before and at the end of a heat wave, measured every two hours at 10 and 20 cm depth, in the irrigated vineyard; the red line indicates the air temperature.

Irrigation is probably the most important and effective short-term adaptation strategy to face the impacts of climate change in Mediterranean vineyards, attending to its high effectiveness in moderating thermal microclimatic extremes at both soil, plant and atmosphere levels (Andrade et al., 2014; Costa et al., 2016) (see **Figures 3**, **5**). Watering and higher soil moisture promote transpiration and the related evaporative cooling in plants, and also favor soil water evaporation (**Figures 1**, **3**). As a result, irrigation in vineyards expanded fast in southern Europe (Costa et al., 2016; Fraga et al., 2018; Gambetta et al., 2021), but water resources are increasingly scarce and demand more precise irrigation management in Mediterranean vineyards (Mirás-Avalos and Araujo, 2021).

**Figure 6:**
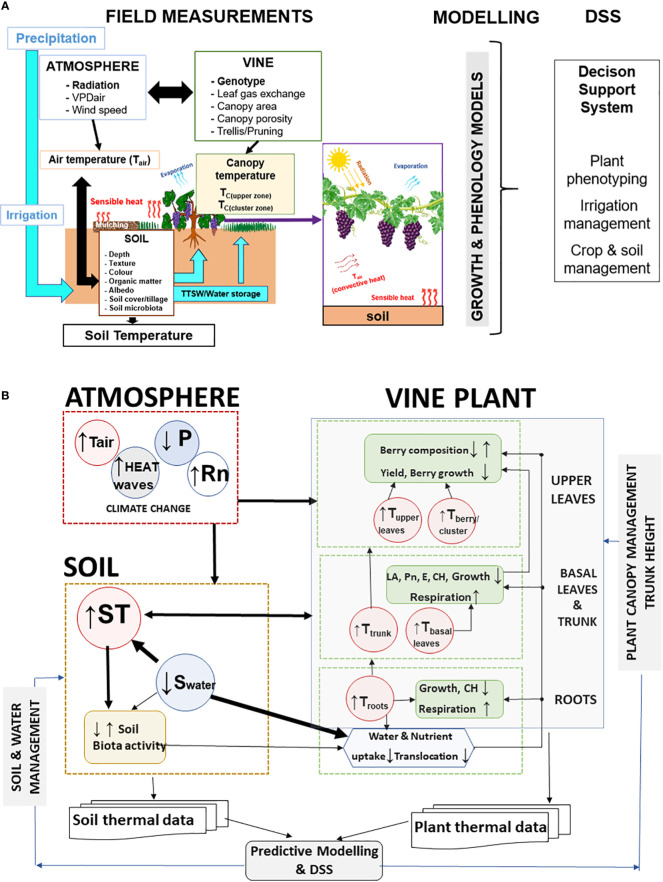
**(A)** Diagram illustrating the potential interactions between soil, canopy and berries in terms of heat exchange and temperature regulation and the use of air temperature (T_air_), soil temperature (TS), canopy temperature (T_C_), and cluster or berry temperature as parameters to feed models to support decision on plant phenotyping, and vineyard management (irrigation, canopy and soil). TTSW – total transpirable soil water; VPDair - Air Vapour-Pressure Deficit (Adapted from Costa et al., 2020). **(B)** Conceptual framework showing the interaction between soil temperature (ST) and soil water (Swater) on vines temperature (roots, trunk, basal and upper leaves, berry/cluster) in a context of climate change (higher radiation -Rn, air temperature -Tair, less precipitation - P). Atmospheric conditions and ST influence vine plants T and morpho-physiological and biophysical processes (Leaf area - LA, Net photosynthesis - Pn, Transpiration - E, Carbohydrates - CH, Soil temperature - ST) depends on atmospheric climate conditions (Rn, Tair, P). Higher Rn and Tair and lower P, promote soil warming and increase evapotranspiration. Soil and plant management influence the thermal regulation of both soil and plants and can be optimized by thermal data (atmosphere, plant and soil) and Decision Support Systems - DSS.

A detailed soil characterization (soil profile, soil properties, fertility) (**Figure 4**) in new vineyards and in the already installed ones is crucial to support more efficient irrigation and fertilization programs. Soil characterization is also essential for an effective distribution of soil water sensors across the vineyard. In addition, thermal measurements of soil, air and plants (punctual and image-based) coupled with computer–based information systems can support Decision Support Systems (DSS) (**Figure 6**) for more efficient vineyard management (Costa et al., 2020; Naulleau et al., 2021). DSS systems using these multi-parameter thermal data besides supporting precise irrigation strategies are potential indicators of water and heat stress in vineyards that can help to predict and mitigate climate risks.

In addition to irrigation, ST (and plant temperature) can be regulated in vineyards under warmer and dryer conditions, by promoting the use of spontaneous soil cover vegetation, selected cover crops, or mulching. Soil cover protects against soil erosion, increases infiltration and water retention, reduces evaporation, and in the case of living mulches or maintenance of adequate spontaneous vegetation, they act as a source of nutrients and OM, and can improve physical, chemical and biological conditions (Marshall et al., 1996; Lazcano et al., 2020). Specifically, natural or spontaneous vegetation cover can also stimulate deeper vine root distribution and promote the use of resources in deeper soil layers (Pradel and Pieri, 2000). The replacement of mineral fertilizers and herbicides with cover crops or vegetation will take years to have a proper impact on soil nutrients and microbial activity, apart from the need to monitor and maintain soil cover (Döring et al., 2019).

Mulching can help to control pests and weeds and maintain yield levels under adverse climatic conditions. Fraga and Santos (2018) investigated the effects of mulching in a typical Mediterranean climate region in Southern Portugal, under future climate change scenarios. Although ST was not directly addressed, their results suggest that mulch can mitigate the adverse effects of hotter and drier weather and extreme events, expressed by an estimated increase of yield by 10 to 25% as compared to bare soil vineyards. The use of soil covering material (mulches) influences maximum summer ST, minimum winter ST, as well as the daily ST fluctuations. In apple orchards, for example, plastic mulching promoted more extreme maximum and minimum ST, but the effect on weed control and water losses was positive and resulted in no major negative effects on plants’ performance (Neilsen et al., 1986).

A more sustainable soil management involving no-tillage or improved tillage strategies is key to minimize soil erosion, decrease soil compaction and avoid the formation of impermeable layers which influence soil thermal and water regimes, nutrient cycles and crop performance. Tillage strategies must be based on a good spatial characterization of the soil profile and properties, avoiding the numerous drawbacks of its use and at larger spatial scales, as it hampers surface water run-off, increases greenhouse gas emissions, difficult the groundwater recharge and promote biodiversity losses (Gürsoy, 2021) **(** **Figure 4** **)**.

Adaptation to increasing ST may encompass larger rooting depth and involve the use of rootstocks with a wider root-zone temperature optimum to enhance the future performance of woody perennial crops (Koevoets et al., 2016; Darriaut et al., 2022). Therefore, selecting new rootstocks to specific environments should be a challenge to face cultivation problems associated with global climate change.

Other strategies to minimize soil and canopy insulation, control ST and protect crops from light stress and high temperatures may be envisaged. However, they are costly and/or may have negative environmental impacts (e.g. visual pollution; recycling issues). This is the case of the use of shading nets in VSP trellis systems as a strategy to mitigate the negative impact of heat waves and sun exposure of berries. Indeed, partial shading (less than 60% of solar radiation) at the cluster zone reduced by about 4 °C the cluster temperature as compared to sunlit clusters (Marigliano et al., 2022). Shadowing in combination with water availability can avoid berry dehydration during the last phases of ripening with positive effects on anthocyanins and flavonols, as compared to sun exposed clusters (Martínez-Lüscher et al., 2020; Marigliano et al., 2022). Nowadays, the installation of photovoltaic panels over crops (“agrivoltaic” farming) is being advertised as a win-win solution for climate change adaptation of vineyards and to produce energy (Frauhnofer, 2022). Nevertheless, vine’s morpho-physiological responses must be taken into account and the respective cost-benefits analysis must be carried out, along with the quantification of the damaging effects on the landscape (e.g. loss of the aesthetics of the natural rural landscapes). Furthermore, row orientation has a dramatic effect on the vine’s exposition to sunlight and consequently on ST and canopy temperature (White, 2015; Hunter et al., 2020; Pisciotta et al., 2021). In addition, row spacing, and trellis design influence ST by varying the percentage of shading of solar radiation on the soil. For example, the soil layers of east – west oriented rows reach their highest temperature in the afternoon, and ST generally increases in the two top layers and decreases in the lower layers from mid-morning to late afternoon (Hunter et al., 2020). A combination of higher planting density with shading can also be considered, which favors both natural and artificial shading in vineyards and minimizes the impacts of extreme radiation and heat conditions on both crops and soil cover crops and benefit the activity of the relevant soil micro-organisms (microbiota).

## 6 Conclusions

More sustainable agricultural, hydrological, and environmental management in the context of climate change demands a better understanding of soil resources variability, at increasingly higher resolutions (Wulf et al., 2014). Though soil temperature maps are already available for many regions of the world (Lembrechts et al., 2022), high resolution data on ST that can be representative of microhabitat conditions for below-ground organisms is still needed, and especially for deeper soil layers.

The effects of spatiotemporal variation of temperature on ecological processes and functioning of agroecosystems has been investigated but the predictive capacity remains low, and more studies focused on the interaction of soil-organisms-crop productivity and quality are still required (Pipan et al., 2021). This knowledge at fine scales would help to better understand the roles of soil and soil management on climate change adaptation and will help to cope with current and future challenges of climate change by supporting predictive modelling and decision-making applied to perennial crops systems, such as grapevine or other typical Mediterranean crops

Long-term field measurements using sensors of both ST and soil moisture are being developed and tested in vineyards and other perennial crops (Garcia-Tejero et al., 2018; Costa et al., 2019; Wild et al., 2019). Thermal sensors have become less expensive and offer larger robustness and energy autonomy though limitations such the low contact of the logger in certain soil types (e.g. drying clay soils) were reported (Wild et al., 2019).

Climate warming may have diverse effects on ST according to the diverse types of heat stress (heat shocks, heat waves, or increasing warming conditions), leading to diverse physiological and molecular responses at leaf and fruit levels, and on root morphology as well as on reproductive traits. There is evidence that the phenological stage of crops influences crops vulnerability to increase temperature, either by pulses or in a continuous trend (Jagadish et al., 2021). This applies to grapevines. Future research should encompass a better understanding of the mechanism(s) by which ST affects leaf and berry traits across different grapevine varieties, clones and/or rootstocks.

Soil remains sidelined in viticulture research, suggesting a lack of attention to non-new but highly relevant issues such as the detailed spatial distribution and characterization of soil types before designing and planting new vineyards. As consequence there is an urgent need to improve monitoring and better evaluate the roles of soil properties and ST in Mediterranean vineyards, which are increasingly exposed to more adverse climatic conditions and increasing irrigation limitations (Costa et al., 2016). A better understanding of the roles of soil properties on soil microbiota, weed and vine morpho-physiology, and on heat and water fluxes, is crucial to achieve a more efficient soil and crop management, and to ensure a more sustainable Mediterranean viticulture under extreme stress conditions. This achievement will certainly contribute to the Sustainable Development Goals, namely in terms of soil and biodiversity protection and more sustainable water management. Ultimately, we must consider the feasibility and economic implications of the proposed management strategies that vary with the wine regions and the fact that improved soil and canopy management solutions will be only achieved with multidisciplinary knowledge and more trained professionals.

## Author contributions

JMC: idea, writing, editing, reviewing and funding. MM, FA, AN and RE writing, editing, and reviewing. JMC, FA, PM: illustrations and editing. All authors contributed to the article and approved the submitted version.

## References

[B1] Abad J. Hermoso de Mendoza I. Marín D. Orcaray L. Santesteban L. G. (2021). Cover crops in viticulture. a systematic review (1): Implications on soil characteristics and biodiversity in vineyard. OENO One 55 (1), 295–312. doi: 10.20870/oeno-one.2021.55.1.3599

[B2] Abdel-Ghany A. M. Al-Helal I. M. Alsadon A. Ibrahim A. Shady M. (2022). Measuring and predicting the in-ground temperature profile for geothermal energy systems in the desert of arid regions. Energies 15, 7268. doi: 10.3390/en15197268

[B3] Akter M. Miah M. A. Hassan M. M. Mobin M. N. Baten M. A. (2015). Textural influence on surface and subsurface soil temperature under various conditions. J. Env. Sci. Nat. Resour. 8 (2), 149–154. doi: 10.3329/jesnr.v8i2.26882

[B4] Andrade J. A. Santos F. L. Correia M. Paço T. A. (2014). Effects of irrigation and tree spacing on soil and air temperature profiles of olive orchards. Acta Hortic. 1057, 443–450. doi: 10.17660/ActaHortic.2014.1057.56

[B5] Andrés P. Doblas-Miranda E. Silva-Sánchez A. Mattana S. Font F. (2022). Physical, chemical, and biological indicators of soil quality in Mediterranean vineyards under contrasting farming schemes. Agronomy 12, 2643. doi: 10.3390/agronomy12112643

[B6] Aragüés R. Medina E. T. Clavería I. (2014). Effectiveness of inorganic and organic mulching for soil salinity and sodicity control in a grapevine orchard drip-irrigated with moderately saline waters. instituto nacional de investigación y tecnología agraria y alimentaria (INIA). Spanish J. Agric. Res. 12 (2), 501–508. doi: 10.5424/sjar/2014122-5466

[B7] Bai E. Li S. Xu W. Li W. Dai W. (2013). A meta-analysis of experimental warming effects on terrestrial nitrogen pools and dynamics. New Phytol. 199, 441–451. doi: 10.1111/nph.12252 23550663

[B8] Basurto-Lozada D. Hillier A. Medina D. Pulido D. Karaman S. (2020). Dynamics of soil surface temperature with unmanned aerial systems. Pattern Recognit. Lett. 138, 68–74. doi: 10.1016/j.patrec.2020.07.003

[B9] Baver L. D. (1965). Soil physics. 3rd ed. (New York: John Wiley & Sons), 489 pp. Fifth Printing.

[B10] Belda I. Ruiz J. Esteban-Fernández A. Navascués E. Marquina D. (2017). Microbial contribution to wine aroma and its intended use for wine quality improvement. Molecules 22 (2), 189. doi: 10.3390/molecules22020189 28125039 PMC6155689

[B11] Bernardo S. Dinis L.-T. Luzio A. Machado N. Gonçalves A. (2021). Optimising grapevine summer stress responses and hormonal balance by applying kaolin in two Portuguese demarcated regions. OENO One 55 (1), 207–222. doi: 10.20870/oeno-one.2021.55.1.4502

[B12] Blankinship J. C. Niklaus P. A. Hungate B. A. (2011). A meta-analysis of responses of soil biota to global change. Oecologia 165 (3), 553. doi: 10.1007/s00442-011-1909-0 21274573

[B13] Bonada M. Jeffery D. W. Petrie P. R. Moran M. A. Sadras V. O. (2015). Impact of elevated temperature and water deficit on the chemical and sensory profiles of barossa Shiraz grapes and wines. Aust. J. Grape Wine Res. 21, 240–253. doi: 10.1111/ajgw.12142

[B14] Bond-Lamberty B. Bailey V. Chen M. Gough C. Vargas R. (2018). Globally rising soil heterotrophic respiration over recent decades. Nature 560, 80–83. doi: 10.1038/s41586-018-0358-x 30068952

[B15] Bosco L. Siegenthaler D. Ruzzante L. Jacot A. Arlettaz R. (2022). Varying responses of invertebrates to biodynamic, organic and conventional viticulture. Front. Conserv. Sci. 3. doi: 10.3389/fcosc.2022.837551

[B16] Bota J. Tomas M. Flexas J. Medrano H. Escalona J. M. (2016). Differences among grapevine cultivars in their stomatal behavior and water use efficiency under progressive water stress. Agric. Water Manage. 164, 91–99. doi: 10.1016/j.agwat.2015.07.016

[B17] Botelho da Costa J. V. (1995). Caracterização e constituição do solo, 5^a^ Edição. 527 pp, com revisão e aditamentos de Ario L. Azevedo e R. Pinto Ricardo. Serviç̧o de Educação, Fundação Calouste Gulbenkian, Lisboa, Portugal. Depósito legal N.° 88 996/95, ISBN 972-31-0073-8.

[B18] Bradford J. B. Schaepfer D. R. Laurenroth W. K. Palmquist K. A. Chambers J. C.. (2019). Climate-driven shifts in soil temperature and moisture regimes suggest opportunities to enhance assessments of dryland resilience and resistance. Front. Ecol. Evol. 7. doi: 10.3389/fevo.2019.00358

[B19] Brady N. C. Weil R. R. (2017). The nature and properties of soils. Fifteenth Edition (New Jersey, USA: Pearson International Edition, Upper Saddle River), 975 pp.

[B20] Bullied W. J. Marginet A. M. Van Acker R. C. (2003). Conventional- and conservation-tillage systems influence emergence periodicity of annual weed species in canola. Weed Sci. 51, 886–897. doi: 10.1614/P2002-117

[B21] Burg P. Čížková A. Mašán V. Sedlar A. Matwijczuk A. Souček J. (2022). The effect of mulch materials on selected soil properties, yield and grape quality in vineyards under central European conditions. Agronomy 12, 1862. doi: 10.3390/agronomy12081862

[B22] Callaham M. A. Jr. Richter D. D. Coleman D. C. Hofmockel M. (2006). Long-term land use effects on soil invertebrate communities in southern piedmont soils. Eur. J. Soil Biol. 42, S150–S156. doi: 10.1016/j.ejsobi.2006.06.001

[B23] Candiago S. Tscholl S. Bassani L. Fraga H. Vigl. L. E. (2022). A geospatial inventory of regulatory information for wine protected designations of origin in Europe. Sci. Data 9, 394. doi: 10.1038/s41597-022-01513-0 35821213 PMC9276794

[B24] Caprio E. Nervo B. Isaia M. Allegro G. Rolando A. (2015). Organic versus conventional systems in viticulture: Comparative effects on spiders and carabids in vineyards and adjacent forests. Agric. Syst. 136, 61–69. doi: 10.1016/j.agsy.2015.02.009

[B25] Carvalho L. C. Coito J. L. Colaço S. Sangiogo M. Amâncio S. (2014). Heat stress in grapevine: the pros and cons of acclimation. Plant Cell. Environ. 38 (4), 778–789. doi: 10.1111/pce.12445 25211707

[B26] Cataldo E. Fucile M. Mattii G. B. (2021). A review: soil management, sustainable strategies and approaches to improve the quality of modern viticulture. Agronomy 11 (11), 2359. doi: 10.3390/agronomy11112359

[B27] Chaves M. M. Costa J. M. Zarrouk O. Pinheiro C. Lopes C. M. Pereira J. S. (2016). Controlling stomatal aperture in semi-arid regions - the dilemma of saving water or being cool? Plant Sci. 251, 54–64. doi: 10.1016/j.plantsci.2016.06.015 27593463

[B28] Chaves M. M. Zarrouk O. Francisco R. Costa J. M. Santos T. Regalado A. P. (2010). Grapevine under deficit irrigation: hints from physiological and molecular data. Ann. Bot. 105, 661–676. doi: 10.1093/aob/mcq030 20299345 PMC2859908

[B29] Clements D. R. Ditommaso A. (2011). Climate change and weed adaptation: can evolution of invasive plants lead to greater range expansion than forecasted? Weed Res. 51, 227–240. doi: 10.1111/j.1365-3180.2011.00850.x

[B30] Compant S. Mitter B. Colli-Mull J. G. Gangl H. Sessitsch A. (2011). Endophytes of grapevine flowers, berries, and seeds: identification of cultivable bacteria, comparison with other plant parts, and visualization of niches of colonization. Microb. Ecol. 62, 188–1197. doi: 10.1007/s00248-011-9883-y 21625971

[B31] Costa J. M. Catarino S. Escalona J. M. Comuzzo P. (2022). “Achieving a more sustainable wine supply chain – environmental and socioeconomic issues of the industry,”. Eds. Costa J. M. Catarino S. Escalona J. M. Comuzzo P. (Academic Press, Elsevier), 1–24, ISBN: 9780323851503. doi: 10.1016/B978-0-323-85150-3.00009-8

[B32] Costa J. M. Egipto R. Lopes C. Silvestre J. (2020). “Using soil and canopy temperature to support efficient management of irrigated vineyards,” in 15th Quantitative InfraRed Thermography (QIRT) conference, Oporto. doi: 10.21611/qirt.2020.120

[B33] Costa J. M. Egipto R. Sánchez-Virosta A. Lopes C. M. Chaves M. M. (2019). Canopy and soil thermal patterns to support water and heat stress management in vineyards. Agric. Water Manage. 216, 484–496. doi: 10.1016/j.agwat.2018.06.001

[B34] Costa J. M. Vaz M. Escalona J. Egipto R. Lopes C. Medrano H. (2016). Modern viticulture in southern Europe: Vulnerabilities and strategies for adaptation to water scarcity. Agric. Water Manage. 164, 5–18. doi: 10.1016/j.agwat.2015.08.021

[B35] Crystal-Ornelas R. Thapa R. Tully K. L. (2021). Soil organic carbon is affected by organic amendments, conservation tillage, and cover cropping in organic farming systems: A meta-analysis. Agric. Ecos. Env. 312, 107356. doi: 10.1016/j.agee.2021.107356

[B36] Culliney T. W. (2013). Role of arthropods in maintaining soil fertility. Agriculture 3, 629–659. doi: 10.3390/agriculture3040629

[B37] Darriaut R. Lailheugue V. Masneuf-Pomarède I. Marguerit E. Martins G.. (2022). Grapevine rootstock and soil microbiome interactions: Keys for a resilient viticulture. Hortic. Res. 9, uhac019. doi: 10.1093/hr/uhac019 35184168 PMC8985100

[B38] Diago M. P. Tardaguila J. Barrio I. Fernández-Novales J. (2022). Combination of multispectral imagery, environmental data and thermography for on-the-go monitoring of the grapevine water status in commercial vineyards. Eur. J. Agron. 140, 126586. doi: 10.1016/j.eja.2022.126586

[B39] Di Giacinto S. Friedel M. Poll C. Döring J. Kunz R. Kauer R.. (2020). Vineyard management system affects soil microbiological properties. OENO One 54 (1), 131–143. doi: 10.20870/oenoone.2020.54.1.2578

[B40] Dong X. Xu W. Zhang Y. Daniel I. Leskovar D. I. (2016). Effect of irrigation timing on root zone soil temperature, root growth and grain yield and chemical composition in corn. Agronomy 6, 34. doi: 10.3390/agronomy6020034

[B41] Döring J. Collins C. Frisch M. Kauer M. (2019). Organic and biodynamic viticulture affect biodiversity and properties of vine and wine: a systematic quantitative review. Am. J. Enol. Vitic. 70 (3), 221–242. doi: 10.5344/ajev.2019.18047

[B42] Droulia F. Charalampopoulos I. (2022). A review on the observed climate change in Europe and its impacts on viticulture. Atmosphere 13, 837. doi: 10.3390/atmos13050837

[B43] Dunn R. R. Agosti D. Andersen A. N. Arnan X. Bruhl C. A. Cerdá X. (2009). Climatic drivers of hemispheric asymmetry in global patterns of ant species richness. Ecol. Lett. 12, 324–333. doi: 10.1111/j.1461-0248.2009.01291.x 19292793

[B44] EEA (2019) Soil, land and climate change. Available at: https://www.eea.europa.eu/signals/signals-2019-content-list/articles/soil-land-and-climate-change.

[B45] Egipto R. Neves M. Mota M. Lopes C. Silvestre J. Costa J. M. (2022) Low-cost sensors as a support tool to monitor soil-plant heat exchanges in a Mediterranean vineyard | TERCLIM, IVES conferences series. Available at: https://ives-openscience.eu/13109/.

[B46] Eisenbeis G. Wichard W. (1987). Atlas on the biology of soil arthropods (Berlin/Heidelberg, Germany: Springer). doi: 10.1007/978-3-642-72634-7

[B47] Escalona J. M. Tomás M. Martorell S. Medrano H. Ribas-Carbo M.. (2012). Carbon balance in grapevines under different soil water supply: importance of whole plant respiration. Aust. J. Grape Wine Res. 18, 308–318. doi: 10.1111/j.1755-0238.2012.00193.x

[B48] Evett S. R. (2000). “Soil physics. 5. energy and water balances at soil-plant-atmosphere interfaces,” in Handbook of soil science. Eds. Sumner M. E. (CRC press), 129–182.

[B49] Feng X. Qian C. Materia S. (2022). Amplification of the temperature seasonality in the mediterranean region under anthropogenic climate. Geoph. Lett. 49 (20), 1–10. doi: 10.1029/2022GL099658

[B50] Fichtl L. Hofmann M. Kahlen K. Voss-Fels K. P. Cast C. S. Ollat N.. (2023). Towards grapevine root architectural models to adapt viticulture to drought. Front. Plant Sci. 14. doi: 10.3389/fpls.2023.1162506 PMC10043487 36998680

[B51] Field S. K. Smith J. P. Morrison E. N. Neil Emery R. J. Holzapfel B. P. (2020). Soil temperature prior to veraison alters grapevine carbon partitioning, xylem sap hormones, and fruit set. Am. J. Enol. Vitic. 71, 52–61. doi: 10.5344/ajev.2019.19038

[B52] Fonseca de Lima C. F. Kleine-Vehn J. De Smet I. Feraru E. (2021). Getting to the root of belowground high temperature responses in plants. J. Exp. Bot. 72 (21), 7404–7413. doi: 10.1093/jxb/erab202 33970267

[B53] Forcella F. (1998). Real-time assessment of seed dormancy and seedling growth for weed management. Seed Sci. Res. 8, 201–210. doi: 10.1017/S0960258500004116

[B54] Fraga H. Cortázar-Atauri I. G. Malheiro A. C. Santos J. A. (2016). Modelling climate change impacts on viticultural yield, phenology and stress conditions in Europe. Glob. Change. Biol. 22, 3774–3788. doi: 10.1111/gcb.13382 27254813

[B55] Fraga H. García de Cortázar Atauri I. Santos J. A. (2018). Viticultural irrigation demands under climate change scenarios in Portugal. Agric. Water Manage. 196, 66–74. doi: 10.1016/j.agwat.2017.10.023

[B56] Fraga H. Morondo M. Leolini L. Santos J. A. (2021). Mediterranean Olive orchards under climate change: A review of future impacts and adaptation strategies. Agronomy 11, 56. doi: 10.3390/agronomy11010056

[B57] Fraga H. Santos J. A. (2018). Vineyard mulching as a climate change adaptation measure: Future simulations for Alentejo, Portugal. Agric. Syst. 164, 107–115. doi: 10.1016/j.agsy.2018.04.006

[B58] Frauhnofer (2022) Agrivoltaics: Opportunities for agriculture and the energy transition a guideline for Germany. Available at: https://www.ise.fraunhofer.de/content/dam/ise/en/documents/publications/studies/APV-Guideline.pdf.

[B59] Frodella W. Lazzeri G. Moretti S. Keizer J. Verheijen F. G. A. (2020). Applying infrared thermography to soil surface temperature monitoring: Case study of a high-resolution 48 h survey in a vineyard (Anadia, Portugal). Sensors 20, 2444. doi: 10.3390/s20092444 32344911 PMC7250030

[B60] Gagnarli E. Goggioli D. Tarchi F. Guidi S. Nannelli R. Vignozzi N.. (2015). Case study of microarthropod communities to assess soil quality in managed vineyards. Soil 1, 527–536. doi: 10.5194/soil-1-527-2015

[B61] Gago J. Estrany J. Estes L. Fernie A. R. Alorda B. Brotman Y.. (2020). Nano and micro unmanned aerial vehicles (UAVs): A new grand challenge for precision agriculture? Curr. Prot. Plant Biol. 5 (1), e20103. doi: 10.1002/cppb.20103 32074410

[B62] Galat Giorgi E. Keller M. Sadras V. Alejandro-Roig F. Perez-Peña J. (2020). High temperature during the budswell phase of grapevines increases shoot water transport capacity. Agric. For. Met. 295, 108173. doi: 10.1016/j.agrformet.2020.108173

[B63] Gambetta J. M. Holzapfel B. P. Stoll M. Friedel M. (2021). Sunburn in grapes: A review. Front. Plant Sci. 11. doi: 10.3389/fpls.2020.604691 PMC7819898 33488654

[B64] Garcia L. Celette F. Gary C. Ripoche A. Valdés-Gómez H. Metay (2018). Management of service crops for the provision of ecosystem services in vineyards: A review. Agric. Eco. Env. 251, 158–170. doi: 10.1016/j.agee.2017.09.030

[B65] Garcia-Tejero I. F. Ortega-Arévalo C. J. Iglesias-Contreras M. Moreno J. M. Souza L.. (2018). Assessing the crop-water status in almond (*Prunus dulcis* mill.) trees *via* thermal imaging camera connected to smartphone. Sensors 18 (4), 1050. doi: 10.3390/s18041050 29614740 PMC5948678

[B66] Gavelienė V. Jurkonienė S. Jankovska-Bortkevič E. Švegždienė D. (2022). Effects of elevated temperature on root system development of two lupine species. Plants 11 (2), 192. doi: 10.3390/plants11020192 35050080 PMC8777784

[B67] Ghiglieno I. Simonetto A. Donna P. Tonni M. Valenti L. Bedussi F.. (2019). Soil biological quality assessment to improve decision support in the wine sector. Agronomy 9, 593. doi: 10.3390/agronomy9100593

[B68] Ghiglieno I. Simonetto A. Orlando F. Donna P. Tonni M.. (2020). Response of the arthropod community to soil characteristics and management in the franciacorta viticultural area (Lombardy, Italy). Agronomy 10 (5), 740. doi: 10.3390/agronomy10050740

[B69] Giffard B. Winter S. Guidoni S. Nicolai A. Castaldini M. Cluzeau D.. (2022). Vineyard management and its impacts on soil biodiversity, functions, and ecosystem services. Front. Ecol. Evol. 10. doi: 10.3389/fevo.2022.850272

[B70] Gobbi A. Acedo A. Imam N. Santini R. G. Ortiz-álvarez R. Ellegaard-Jensen L.. (2022). A global microbiome survey of vineyard soils highlights the microbial dimension of viticultural terroirs. Commun. Biol. 5, 241. doi: 10.1038/s42003-022-03202-5 35304890 PMC8933554

[B71] Gonçalves F. Carlos C. Crespo L. Zina V. Oliveira A. Salvação S.. (2021). Soil arthropods in the douro demarcated region vineyards: General characteristics and ecosystem services provided. Sustainability 13, 7837. doi: 10.3390/su13147837

[B72] Greer D. H. Weedon M. M. (2012). Modelling photosynthetic responses to temperature of grapevine (Vitis vinifera cv. semillon) leaves on vines grown in a hot climate. Plant Cell Env. 35 (6), 1050–1064. doi: 10.1111/j.1365-3040.2011.02471.x 22150771

[B73] Griffiths R. P. Madritch M. D. Swanson A. K. (2009). The effects of topography on forest soil characteristics in the Oregon cascade mountains (USA): Implications for the effects of climate change on soil properties. For. Ecol. Manage. 257, 1–7. doi: 10.1016/j.foreco.2008.08.010

[B74] Guerra J. G. Cabello F. Fernández-Quintanilla C. Peña J. M. Dorado J. (2022). How weed management influence plant community composition, taxonomic diversity and crop yield: A long-term study in a Mediterranean vineyard. Agric. Eco. Env. 326, 107816. doi: 10.1016/j.agee.2021.107816

[B75] Guion A. Turquety S. Polcher J. Pennel R. Bastin S. Arsouze T. (2022). Droughts and heatwaves in the Western Mediterranean: impact on vegetation and wildfires using the coupled WRF-ORCHIDEE regional model (RegIPSL). Clim. Dyn. 58, 2881–2903. doi: 10.1007/s00382-021-05938-y

[B76] Guoju X. Qiang Z. Jiangtao B. Fengju Z. Chengke L. (2020). The relationship between winter temperature rise and soil fertility properties. Air Soil Water Res. 5. doi: 10.1177/ASWR.S8599

[B77] Gürsoy S. (2021). “Soil compaction due to increased machinery intensity in agricultural production: its main causes, effects and management,” in Technology in agriculture. Eds. Ahmad F. Sultan M. (IntechOpen). doi: 10.5772/intechopen.98564

[B78] Gutiérrez S. Diago M. P. Fernández-Novales J. Tardaguila J. (2018). Vineyard water status assessment using on-the-go thermal imaging and machine learning. PLoS One 13 (2), e0192037. doi: 10.1371/journal.pone.0192037 29389982 PMC5794144

[B79] Haddaway N. R. Hedlund K. Jackson L. E. Katterer T. Lugato E.. (2017). How does tillage intensity affect soil organic carbon? a systematic review. Environ. Evidence 6, 30. doi: 10.1186/s13750-017-0108-9

[B80] Heilman J. L. McInnes K. J. Savage M. J. Gesh R. W. Lascano R. J. (1994). Soil and canopy energy balances in a west Texas vineyard. Agric. For. Met. 71, 99–114. doi: 10.1016/0168-1923(94)90102-3

[B81] Hendrix P. F. (2000). “Soil fauna,” in Handbook of soil science. Ed. Sumner M. E. (Boca Raton, FL, USA: CRC Press).

[B82] Hillel D. (2004). Introduction to environmental soil physics (Amsterdam: Elsevier, Academic Press), 494 pp.

[B83] Hirschi M. Seneviratne S. Alexandrov V. Boberg F. Boroneant C. (2011). Observational evidence for soil-moisture impact on hot extremes in south-eastern Europe. Nat. Geosci. 4, 17–21. doi: 10.1038/ngeo1032

[B84] Houle D. Bouffard A. Duchesne L. Logan T. Harvey R. (2012). Projections of future soil temperature and water content for three southern Quebec forested sites. J. Climate 25 (21), 7690–7701. doi: 10.1175/JCLI-D-11-00440.1

[B85] Howell A. Winkler D. E. Phillips M. L. McNellis B. Reed S. C. (2020). Experimental warming changes phenology and shortens growing season of the dominant invasive plant bromus tectorum (cheatgrass). Front. Plant Sci. 11. doi: 10.3389/fpls.2020.570001 PMC7593257 33178240

[B86] Hu H. W. Macdonald C. A. Trivedi P. Anderson I. C. Zheng Y.. (2016). Effects of climate warming and elevated CO2 on autotrophic nitrification and nitrifiers in dryland ecosystems. Soil Biol. Biochem. 92, 1–5. doi: 10.1016/j.soilbio.2015.09.008

[B87] Huang X. Lakso A. N. Eissenstat D. M. (2005). Interactive effects of soil temperature and moisture on concord grape root respiration. J. Exp. Bot. 56, 2561–2660. doi: 10.1093/jxb/eri258 16143721

[B88] Hunter J. J. K. Tarricone L. Volschenk C. Giacalone C. Melo M. S. (2020). Grapevine physiological response to row orientation-induced spatial radiation and microclimate changes. OENO One 54 (2), 411–433. doi: 10.20870/oeno-one.2020.54.2.3100

[B89] Hunter J. J. Volschenk C. G. Mania E. Vicente-Castro A. Booyse M.. (2021). Grapevine row orientation mediated temporal and cumulative microclimatic effects on grape berry temperature and composition. Agric. For. Met. 310, 108660. doi: 10.1016/j.agrformet.2021.108660

[B90] IPCC. (2021). “Climate change 2021: The physical science basis,” in Contribution of working group I to the sixth assessment report of the intergovernmental panel on climate change. Eds. Masson-Delmotte V. Zhai P. Pirani A. Connors S. L. Péan C. (New York, NY: Cambridge University Press).

[B91] Jagadish S. V. K. Way D. A. Sharkey T. D. (2021). Plant heat stress: Concepts directing future research. Plant Cell Environ. 44, 1992–2005. doi: 10.1111/pce.14050 33745205

[B92] Jansson J. K. Hofmockel K. S. (2020). Soil microbiomes and climate change. Nat. Rev. Microbiol. 18, 35–46. doi: 10.1038/s41579-019-0265-7 31586158

[B93] Jones G. (2012). “A climate assessment for the douro wine region: An examination of the past, present and future climate conditions for wine production,” in ADVID – associação para o desenvolvimento da viticultura duriense (Régua: Godim).

[B94] Jones H. G. Stoll M. Santos T. Sousa C. Chaves M. M. Grant O. M. (2002). Use of infrared thermography for monitoring stomatal closure in the field: Application to grapevine. J. Exp. Bot. 53 (378), 2249–2260. doi: 10.1093/jxb/erf083 12379792

[B95] Jones H. G. Vaughan A. R. (2010). Remote sensing of vegetation: principles, techniques, and applications. Oxford University Press, New York. 353.

[B96] Karhu K. Auffret M. Dungait J. Hopkins D. Prosser J. Singh B. K.. (2014). Temperature sensitivity of soil respiration rates enhanced by microbial community response. Nature 513, 81–84. doi: 10.1038/nature13604 25186902

[B97] Kathiresan R. Gualbert G. (2016). Impact of climate change on the invasive traits of weeds. Weed Biol. Manage. 16, 59–66. doi: 10.1111/wbm.12096

[B98] Keller M. Scheele-Baldinger R. Ferguson J. C. Tarara J. M. Mills L. J. (2022). Inflorescence temperature influences fruit set, phenology, and sink strength of Cabernet sauvignon grape berries. Front. Plant Sci. 13. doi: 10.3389/fpls.2022.864892 PMC9420974 36046582

[B99] Koevoets I. T. Venema J. H. Elzenga J. T. M. Testerink C. (2016). Roots withstanding their environment: exploiting root system architecture responses to abiotic stress to improve crop tolerance. Front. Plant Sci. 7. doi: 10.3389/fpls.2016.01335 PMC5005332 27630659

[B100] Krapez J. C. Chatelard C. Nouvel J. F. Déliot Ph. (2012). “Combined airborne thermography and visible-to-near infrared reflectance measurement for soil moisture mapping,” in Proceedings of the 11th International Conference on Quantitative InfraRed Thermography (QIRT 2012), Naples, Italy, Vol. 17. 11–14, june. e-J. Nondest. Testing. doi: 10.21611/qirt.2012.231

[B101] Kustas W. P. Nieto H. Garcia-Tejera O. Bambach N. McElrone A. J. (2022). Impact of advection on two-source energy balance (TSEB) canopy transpiration parameterization for vineyards in the California central valley. Irrig Sci. 40, 575–591. doi: 10.1007/s00271-022-00778-y

[B102] Lanyon D. M. Cass A. Hansen D. (2004). The effect of soil properties on vine performance. CSIRO Land Water. Technical Report No. 34/04. doi: 10.4225/08/586be7e218029

[B103] Lazcano C. Decock C. Wilson S. G. (2020). Defining and managing for healthy vineyard soils intersection, with the concept of terroir. Front. Environ. Sci. 8. doi: 10.3389/fenvs.2020.00068

[B104] Lembrechts J. J. van den Hoogen J. Aalto J. Ashcroft M. B. De Frenne P. (2022). Global maps of soil temperature. Global Change Biol. 28, 3110–3144. doi: 10.1111/gcb.16060 PMC9303923 34967074

[B105] Li M. Wu P. Ma Z. (2020). A comprehensive evaluation of soil moisture and soil temperature from third-generation atmospheric and land reanalysis data sets. Int. J. Climat. 40 (13), 5744–5766. doi: 10.1002/joc.6549

[B106] Liu S. Li J. Zhang X. (2022). Simulations of soil water and heat processes for no tillage and conventional tillage systems in mollisols of China. Land 11, 417. doi: 10.3390/land11030417

[B107] Lopes C. M. Santos T. P. Rodrigues M. L. Monteiro A. Costa J. M. (2011). Combining cover cropping with deficit irrigation in a Mediterranean low vigor vineyard. Sci. Hortic. 129, 603–612. doi: 10.1016/j.scienta.2011.04.033

[B108] Lorenz R. Jaeger E. B. Seneviratne S. I. (2010). Persistence of heat waves and its link to soil moisture memory. Geoph. Res. Lett. 37, L09703. doi: 10.1029/2010GL042764

[B109] Luo H. Xu H. Chu C. He F. Fang S. (2020). High temperature can change root system architecture and intensify root interactions of plant seedlings. Front. Plant Sci. 11. doi: 10.3389/fpls.2020.00160 PMC7054236 32161613

[B110] Mancuso S. Shabala S. (Eds.) (2010). Waterlogging signalling and tolerance in plants (Heidelberg: Springer). doi: 10.1007/978-3-642-10305-6

[B111] Marigliano L. E. Yu R. Torres N. Tanner J. D. Battany M. (2022). Photoselective shade films mitigate heat wave damage by reducing anthocyanin and flavonol degradation in grapevine (*Vitis vinifera* l.) berries. Front. Agron. 4. doi: 10.3389/fagro.2022.898870

[B112] Marshall T. J. Holmes J. W. Rose C. W. (1996). Soil physics. 3rd ed. (Cambridge University Press), 453 pp.

[B113] Martínez-Lüscher J. Chen C. C. L. Brillante L. Kurtural S. K. (2020). Mitigating heat wave and exposure damage to “Cabernet sauvignon” wine grape with partial shading under two irrigation amounts. Front. Plant Sci. 11 (November). doi: 10.3389/fpls.2020.579192 PMC7683524 33240297

[B114] Medrano H. Perez-Peña J. Prieto J. Tomás M. Franck N. Escalona J. M. (2016). “Carbon balance in grapevine under a changing climate,” in Grapevine in a changing environment: A molecular and ecophysiological PerspectiveEdition. Eds. Varanda Gerós H. Chaves M. M. Medrano H. Delrot S. (John Wiley & Sons, Ltd.), pp.110–pp.134. doi: 10.1002/9781118735985.ch5

[B115] Mehdizadeh S. Ahmadi F. Sales A. K. (2020a). Modelling daily soil temperature at different depths via the classical and hybrid models. Met. Appl., 1–15. doi: 10.1002/met.1941

[B116] Mehdizadeh S. Fathian F. Safari M. J. S. Khosravi A. (2020b). Developing novel hybrid models for estimation of daily soil temperature at various depths. Soil Till. Res. 197, 104513. doi: 10.1016/j.still.2019.104513

[B117] Meinhold T. Richters J.-P. Damerow L. Blanke M. M. (2010). ‘Optical properties of reflection ground covers with potential for enhancing fruit colouration’. Biosystem Eng. 107 (2), 155–160. doi: 10.1016/j.biosystemseng.2010.07.006

[B118] Melillo J. M. Frey S. D. Deangelis K. M. Werner W. J. Bernard M. J. Bowles F. P.. (2017). Long-term pattern and magnitude of soil carbon feedback to the climate system in a warming world. Science 358, 101–104. doi: 10.1126/science.aan2874 28983050

[B119] Mirás-Avalos J. Araujo E. S. (2021). Optimization of vineyard water management: Challenges, strategies, and perspectives. Water 13, 746. doi: 10.3390/w13060746

[B120] Moutinho-Pereira J. M. Correia C. M. Gonçalves B. M. Bacelar E. A. Torres-Pereira J. M. (2004). Leaf gas exchange and water relations of grapevines grown in three different conditions. Photosynthetica 42 (1), 81–86. doi: 10.1023/B:PHOT.0000040573.09614.1d

[B121] Nagel K. A. Kastenholz B. Jahnke S. van Dusschoten D. Aach T. Mühlich M.. (2009). Temperature responses of roots: impact on growth, root system architecture and implications for phenotyping. Funct. Plant Biol. 36 (11), 947–959. doi: 10.1071/FP09184 32688706

[B122] Naulleau A. Gary C. Prévot L. Hossard L. (2021). Evaluating strategies for adaptation to climate change in grapevine production–a systematic review. Front. Plant Sci. 11. doi: 10.3389/fpls.2020.607859 PMC7840846 33519859

[B123] Neilsen G. H. Hogue E. J. Drought B. G. (1986). The effect of orchard soil management on soil temperature and apple tree nutrition. Can. J. Soil Sci. 66, 701–711. doi: 10.4141/cjss86-069

[B124] Nobel P. S. (2005). Physicochemical and environmental plant physiology (San Diego: Elsevier/ Academic Press).

[B125] Nóia Júnior R. S. do Amaral G. C. Pezzopane J. E. M. Toledo J. V. Xavier T. M. T. (2018). Ecophysiology of C3 and C4 plants in terms of responses to extreme soil temperatures. Theor. Exp. Plant Physiol. 30, 261–274. doi: 10.1007/s40626-018-0120-7

[B126] OIV (2022) State of the world vine and wine sector 2021. Available at: https://www.oiv.int/sites/default/files/documents/eng-state-of-the-world-vine-and-wine-sector-april-2022-v6_0.pdf.

[B127] Onofre J. (2022). “European Wine policy framework - the path toward sustainability,” in Improving sustainable viticulture and winemaking practices. Eds. Costa J. M. Catarino S. Escalona J. Commuzzo P. (Academic Press, Elsevier), 485–499.

[B128] Onwuka B. Mang B. (2018). Effects of soil temperature on some soil properties and plant growth. review article. Adv. Plants Agric. Res. 8 (1), 34–37. doi: 10.15406/apar.2018.08.00288

[B129] Pagay V. Collins C. (2017). Effects of timing and intensity of elevated temperatures on reproductive development of field-grown Shiraz grapevines. OENO One 51 (4), 409–421. doi: 10.20870/oeno-one.2017.51.4.1066

[B130] Paiola A. Assandri G. Brambilla M. Zottini M. Pedrini P.. (2020). Exploring the potential of vineyards for biodiversity conservation and delivery of biodiversity-mediated ecosystem services: A global-scale systematic review. Sci. Total Env. 706, 135839. doi: 10.1016/j.scitotenv.2019.135839 31846877

[B131] Parolin P. Scotta M. Bresch C. (2014). Biology of dittrichia viscosa, a Mediterranean ruderal plant: a review. Intern. J. Exp. Bot. 83, 251–262. doi: 10.32604/phyton.2014.83.251

[B132] Pierce S. Maffi D. Faoro F. Cerabolini B. E. L. Spada A. (2022). The leaf anatomical trade-offs associated with plant ecological strategy variation. Plant Ecol. 223, 1233–1246. doi: 10.1007/s11258-022-01270-5

[B133] Pinto C. Pinho D. Sousa S. Pinheiro M. Egas C. (2014). Unravelling the diversity of grapevine microbiome. PLoS One 9 (1), e85622. doi: 10.1371/journal.pone.0085622 24454903 PMC3894198

[B134] Pipan P. Hall A. Rogiers S. Y. Holzapfel B. P. (2021). Accuracy of interpolated versus in-vineyard sensor climate data for heat accumulation modelling of phenology. Front. Plant Sci. 12. doi: 10.3389/fpls.2021.635299 PMC8313810 34326852

[B135] Pisciotta A. Catania P. Orlando S. Vallone M. (2021). Influence of row orientation on the canopy temperature of Sicilian vineyards. Acta Hortic. 1314, 367–374. doi: 10.17660/ActaHortic.2021.1314.46

[B136] Pradel E. Pieri P. (2000). Influence of a grass layer on vineyard soil temperature. Aust. J. Grape Wine Res. 6 (1), 59–67. doi: 10.1111/j.1755-0238.2000.tb00163.x

[B137] Pritchard S. G. (2011). Soil organisms and global climate change. Plant Pathol. 60, 82–99. doi: 10.1111/j.1365-3059.2010.02405.x

[B138] Radke J. K. (1982). Managing early season soil temperatures in the northern corn belt using configured soil surfaces and mulches. Soil Sci. Soc J. Am. 46, 1067–1071. doi: 10.2136/sssaj1982.03615995004600050036x

[B139] Reddy M. V. Venkataiah B. (1990). Seasonal abundance of soil-surface arthropods in relation to some meteorological and edaphic variables of the grassland and tree-planted areas in a tropical semi-arid savanna. Int. J. Biometeorol. 34, 49–59. doi: 10.1007/BF01045820

[B140] Rességuier L. Pieri P. Mary S. Pons R. Petijean T.. (2023). Characterisation of the vertical temperature gradient in the canopy reveals increased trunk height to be a potential adaptation to climate change. OENO One 57 (1), 41–53. doi: 10.20870/oeno-one.2023.57.1.5365

[B141] Rogiers S. Y. Smith. J. P. Holzapfel. B. P. Hardie W. J. (2011). Soil temperature moderates grapevine carbohydrate reserves after bud break and conditions fruit set responses to photoassimilatory stress. Funct. Plant Biol. 38 (11), 899–909. doi: 10.1071/FP10240 32480947

[B142] Romero-Olivares A. L. Allison S. D. Treseder K. K. (2017). Soil microbes and their response to experimental warming over time: a meta-analysis of field studies. Soil Biol. Biochem. 107, 32–40. doi: 10.1016/j.soilbio.2016.12.026

[B143] Ruperti B. Botton A. Populin F. Eccher G. Brilli M.. (2019). Flooding responses on grapevine: A physiological, transcriptional, and metabolic perspective. Front. Plant Sci. 10. doi: 10.3389/fpls.2019.00339 PMC6443911 30972087

[B144] Samouëlian A. Cousin I. Tabbagh A. Bruand A. Richarde G. (2005). Electrical resistivity survey in soil science: a review. Soil Till. Res. 83 (2), 173–193. doi: 10.1016/j.still.2004.10.004

[B145] Santos J. A. Fraga H. Malheiro A. C. Moutinho-Pereira J. Dinis L.-T.. (2020). A review of the potential climate change impacts and adaptation options for European viticulture. Appl. Sci. 10, 3092. doi: 10.3390/app10093092

[B146] Sassenrath G. F. Davis K. Sassenrath-Cole A. Riding N. (2018). Exploring the physical, chemical and biological components of soil: Improving soil health for better productive capacity. Kansas Agric. Experiment Station Res. Rep. 4 (3), 1–8. doi: 10.4148/2378-5977.7577

[B147] Sauer J. Struik G. (1964). A possible ecological relation between soil disturbance, light-flash, and seed germination source. Ecology 45, 884–886. doi: 10.2307/1934942

[B148] Schindlbacher A. Rodler A. Kuffner M. Kitzler B. Sessitsch A.. (2011). Experimental warming effects on the microbial community of a temperate mountain forest soil. Soil Biol. Biochem. 43 (7), 1417–1425. doi: 10.1016/j.soilbio.2011.03.005 21760644 PMC3103824

[B149] Schultz H. (2022). Soil, vine, climate change; the challenge of predicting soil carbon changes and greenhouse gas emissions in vineyards and is the 4 per 1000 goal realistic? OENO One 56 (2), 251–263. doi: 10.20870/oeno-one.2022.56.2.5447

[B150] Schwarz K. Heil J. Marschner B. Stumpe B. (2021). Hot movements on soil surfaces – innovative insights into microbial dynamics using passive infrared thermography. Geoderma 385, 114879. doi: 10.1016/j.geoderma.2020.114879

[B151] Seneviratne S. I. Corti T. Davin E. L. Hirschi M. Jaeger E. B. Lehner I.. (2010). Investigating soil moisture–climate interactions in a changing climate: A review. Earth Sci. Rev. 99 (3–4), 125–161. doi: 10.1016/j.earscirev.2010.02.004

[B152] Shah A. M. Khan I. M. Shah T. I. Bangroo S. A. Kirmani N. A. Nazir S.. (2022). Soil microbiome: a treasure trove for soil health sustainability under changing climate. Land 11 (11), 1887. doi: 10.3390/land11111887

[B153] Simonneau T. Lebon E. Coupel-Ledru A. Marguerit E. Rossdeutsch L. Ollat N.. (2017). Adapting plant material to face water stress in vineyards: which physiological targets for an optimal control of plant water status? OENO One 51 (2), 167–179. doi: 10.20870/oeno-one.2017.51.2.1870

[B154] Snyder B. A. Callaham M. A. (2019). “Soil fauna and their potential responses to warmer soils,” in Ecosystem consequences of soil warming: Microbes, vegetation, fauna and soil biogeochemistry. Ed. Mohan J. E. (Elsevier), 279–296.

[B155] Soil Survey Staff (2014). Keys to soil taxonomy. 13th edn (Washington, DC: NRCS, USDA).

[B156] Sremac A. F. Lalic B. Cuxart J. Marcic M. (2021). Maximum, minimum, and daily air temperature range in orchards: what do observations reveal? Atmosphere 12, 10, 1279. doi: 10.3390/atmos12101279

[B157] Stéfanon M. Drobinski P. D’Andrea F. Lebeaupin-Brossier C. Bastin S. (2014). Soil moisture-temperature feedbacks at meso-scale during summer heat waves over western Europe. Clim. Dyn. 42 (5–6), 1309–1324. doi: 10.1007/s00382-013-1794-9

[B158] Tadayon M. S. Hossein S. M. (2022). Shade net and mulching measures for improving soil and plant water status of fig trees under rainfed conditions. Agric. Water Manage. 271, 107796. doi: 10.1016/j.agwat.2022.107796

[B159] Tombesi S. Cincera I. Frioni T. Ughini V. Gatti M. Palliotti A.. (2019). Relationship among night temperature, carbohydrate translocation and inhibition of grapevine leaf photosynthesis. Environ. Exp. Bot. 157, 293–298. doi: 10.1016/j.envexpbot.2018.10.023

[B160] Van Leeuwen C. Destrac-Irvine A. (2017). Modified grape composition under climate change conditions requires adaptations in the vineyard. OENO One 51, 2. doi: 10.20870/oeno-one.2017.51.2.1647

[B161] Van Leeuwen C. Roby J.-P. Rességuier L. (2018). Soil related terroir factors, a review. OENO One 52, 2 173–2 188. doi: 10.20870/oeno-one.2018.52.2.2208

[B162] Van Leeuwen C. Roby J.-P. Rességuier L. (2020). “How to measure and manage the soil effect in terroir expression,” in IVES technical reviews (vine & wine). doi: 10.20870/IVES-TR.2020.4484

[B163] Venios X. Korkas E. Nisiotou A. Banilas G. (2020). Grapevine responses to heat stress and global warming. Plants 9 (12), 1754. doi: 10.3390/plants9121754 33322341 PMC7763569

[B164] Verheijen F. G. A. Jeffery S. Velde M. V. Penížek V. Beland M.. (2013). Reductions in soil surface albedo as a function of biochar application rate: Implications for global radiative forcing. Environ. Res. Lett. 8, 44008. doi: 10.1088/1748-9326/8/4/044008

[B165] Větrovský T. Kohout P. Kopecký M. Machac A. Man M. Bahnmann B. D.. (2019). A meta-analysis of global fungal distribution reveals climate-driven patterns. Nat. Commun. 10, 1–9. doi: 10.1038/s41467-019-13164-8 31723140 PMC6853883

[B166] Vogel J. Paton E. Aich V. (2021). Seasonal ecosystem vulnerability to climatic anomalies in the Mediterranean. Biogeosciences 18, 5903–5927. doi: 10.5194/bg-18-5903-2021

[B167] Wadoux A. McBratney A. (2021). Digital soil science and beyond. Soil Sci. Am. Soil Soc 85 (5), 1313–1331. doi: 10.1002/saj2.20296

[B168] Walker R. Winter E. (2006). “Vine carbohydrate dynamics and source sink relationships,” in Report to grape and wine research and development corporation (CSIRO). Available at: https://www.wineaustralia.com/getmedia/2315d7a1-47c3-4e07-af36-2164883097ff/Final-Reportgwr0202h.

[B169] Wang C. Yang K. (2018). A new scheme for considering soil water-heat transport coupling based on community land model: Model description and preliminary validation. J. Adv. Model. Earth Syst. 10, 927–950. doi: 10.1002/2017MS001148

[B170] White R. (2015). Understanding vineyard soils (Oxford University Press), 263 pp.

[B171] Wild J. Kopecký M. Macek M. Šanda M. Jankovec J. Haase T. (2019). Climate at ecologically relevant scales: A new temperature and soil moisture logger for long-term microclimate measurement. Agric. For. Met. 268, 40–47. doi: 10.1016/j.agrformet.2018.12.018

[B172] Winter S. Bauer T. Strauss P. Kratschmer S. Paredes D. Popescu D.. (2018). Effects of vegetation management intensity on biodiversity and ecosystem services in vineyards: A meta-analysis. J. Appl. Ecol. 55, 2484–2495. doi: 10.1111/1365-2664.13124 30147143 PMC6099225

[B173] Wulf H. Mulder V. L. Schaepman M. Keller A. Joerg P. C. (2014). “Remote sensing of soils,” in Technical report (University of Zurich, INRA). doi: 10.13140/2.1.1098.0649

[B174] Xu C. Qu J. J. Hao X. Zhu Z. Gutenberg L. (2020). Surface soil temperature seasonal variation estimation in a forested area using combined satellite observations and *in-situ* measurements. Int. J. Appl. Earth Observ. Geoinf. Volume 91, 102156. doi: 10.1016/j.jag.2020.102156

[B175] Xyrafis E. G. Fraga H. Nakas C. T. Koundouras S. (2022). A study on the effects of climate change on viticulture on santorini island. OENO One 56, 1. doi: 10.20870/oeno-one.2022.56.1.4843

[B176] Yau I.-H. Davenport J. R. Moyer M. M. (2014). Developing a wine grape site evaluation decision support system for the inland pacific northwestern united states. Hortecnology 24 (1), 88–98. doi: 10.21273/HORTTECH

[B177] Yu O. T. Greenhut R. F. O'Geen A. T. Mackey B. Horwath W. R. Steenwerth K. L.. (2022). Precipitation events, soil type, and vineyard management practices influence soil carbon dynamics in a mediterranean climate (Lodi, California). Soil Sci. Soc Am. J. 83, 772–779. doi: 10.2136/sssaj2018.09.0345

[B178] Yuste J. C. Baldocchi D. D. Gershenson A. Goldstein A. Misson L. Wong S.. (2007). Microbial soil respiration and its dependency on carbon inputs, soil temperature and moisture. Glob. Change Biol. 13, 2018–2035. doi: 10.1111/j.1365-2486.2007.01415

[B179] Zagatto M. R. G. Zanão Júnior L. A. Pereira A. P. A. Estrada-Bonilla G. Nogueira Cardoso E. J. B. (2021). Soil mesofauna in consolidated land use systems: how management affects soil and litter invertebrates. Sci. Agric. 76 (2), 165–171. doi: 10.1590/1678-992X-2017-0139

[B180] Zarraonaindia I. Owens S. M. Weisenhorn P. West K. Hampton-Marcell J.. (2015). The soil microbiome influences grapevine-associated microbiota. mBio 246 (2), e02527–e02514. doi: 10.1128/mBio.02527-14 PMC4453523 25805735

[B181] Zeng H. Tang C.-S. Zhu C. Cheng Q. Lin Z.-Z. Shi B.. (2021). Investigating soil desiccation cracking using an infrared thermal imaging technique. Water Resour. Res. 58, e2021WR030916. doi: 10.1029/2021WR030916

[B182] Zhang W. Parker K. M. Luo Y. Wan S. Wallace L. L. Hu S.. (2005). Soil microbial responses to experimental warming and clipping in a tallgrass prairie. Glob. Change Biol. 11, 266–277. doi: 10.111/j1365-2486.2005.00902.x

[B183] Zhu G. Luo Y. Xue M. Zhao H. Xia N. Wang X.. (2018). Effects of high-temperature stress and heat shock on two root maggots, *Bradysia odoriphaga* and *Bradysia difformis* (Diptera: *Sciaridae*). J. Asia-Pac. Entomol. 21, 106–114. doi: 10.1016/j.aspen.2017.11.001

